# The tumor suppressor FAT1 controls YAP/TAZ protein degradation and tumor cell proliferation through E3 ligase MIB2

**DOI:** 10.1371/journal.pone.0325535

**Published:** 2025-06-06

**Authors:** Rui Li, Boris Strilic, Young-June Jin, Jingchen Shao, Yundong Peng, Lei Wang, Qi Quan, Zhiyong Wang, Johannes Graumann, J. Silvio Gutkind, Nina Wettschureck, Stefan Offermanns

**Affiliations:** 1 Department of Pharmacology, Max Planck Institute for Heart and Lung Research, Bad Nauheim, Germany; 2 Department of Cardiac Development and Remodeling, Max Planck Institute for Heart and Lung Research, Bad Nauheim, Germany; 3 Moores Cancer Center, University of California, San Diego, La Jolla, California, United States of America; 4 Biomolecular Mass Spectrometry Service Group, Max Planck Institute for Heart and Lung Research, Bad Nauheim, Germany; 5 Department of Medicine, Institute of Translational Proteomics, Philipps-University Marburg, Marburg, Germany; 6 Department of Pharmacology, University of California San Diego, La Jolla, California, United States of America; 7 Center for Molecular Medicine, Goethe University Frankfurt, Frankfurt, Germany; 8 Frankfurt Cancer Institute, Goethe University Frankfurt, Frankfurt, Germany; The University of Texas MD Anderson Cancer Center, UNITED STATES OF AMERICA

## Abstract

*FAT1* is a tumor suppressor gene encoding the protocadherin FAT1, which has been found to be mutated in different types of human cancers with the highest frequency in head and neck squamous cell carcinoma (HNSCC). However, through which mechanisms mutations of *FAT1* lead to tumor progression is incompletely understood. Here, we report that loss of FAT1 in various tumor cells, including HNSCC cells, resulted in increased protein levels of the transcriptional regulators YAP and TAZ. This was sufficient to lead to increased expression of YAP/TAZ target genes and to increased tumor cell proliferation. We found that elevated YAP/TAZ activity after loss of FAT1 was due to decreased YAP/TAZ protein degradation, which could be rescued by expression of the intracellular part of FAT1. When analyzing the interactome of the cytoplasmic part of FAT1 in tumor cells, we identified the E3 ubiquitin ligase Mind Bomb-2 (MIB2) as an interaction partner. Suppression of MIB2 expression in various tumor cells led to same effects as loss of FAT1 expression, including a decrease in YAP and TAZ ubiquitination, and degradation as well as an increase in YAP/TAZ protein levels and expression of YAP/TAZ target genes. Similarly, Hela cells or HNSCCs with suppressed MIB2 expression resembled FAT1 defective tumor cells showing faster proliferation *in vitro* as well as increased tumor growth *in vivo* compared to control cells. Our study identifies a mechanism by which YAP/TAZ levels are kept low through FAT1/MIB2-mediated protein degradation and shows that tumor progression resulting from mutation of tumor suppressor FAT1 involves loss of MIB2-dependent degradation of YAP and TAZ.

## Introduction

*FAT1*, which encodes the protocadherin FAT1, has been found to be mutated in various tumors with the highest rate in squamous cell carcinoma [1–6]. *FAT1* acts as a tumor-suppressor gene [7], and loss of FAT1 function induces a hybrid epithelial-to-mesenchymal transition (EMT) phenotype that promotes tumor progression [8]. In various species, FAT1 has been identified as a negative regulator of YAP and TAZ [3,9–11], which are often activated during the growth and progression of tumors [12,13]. YAP and TAZ have been shown to promote tumor cell survival, proliferation, invasive growth, metastasis and resistance to chemotherapy [14–17]. They function as transcriptional cofactors, shuttling between the cytoplasm and nucleus to regulate essential processes such as cell differentiation, proliferation and survival [18–21]. Within the nucleus, YAP and TAZ interact with various transcription factors to regulate gene expression, with the TEAD family being the best studied [22–25].

Loss of FAT1 function in tumor cells results in YAP/TAZ activation, and two major mechanisms have been described how loss of FAT1 function leads to dysregulated YAP/TAZ activity in tumors [2,8]. FAT1 can function as a scaffold to assemble several components of the Hippo signaling pathway [2]. This can trigger the activation of Hippo signaling pathway through the phosphorylation cascade of MST1/2 kinases and subsequently activate LATS1/2 kinases. This leads to the phosphorylation of YAP and TAZ, resulting in their sequestration in the cytoplasm and their eventual degradation [19,21]. Moreover, FAT1 plays a role in preventing YAP/TAZ nuclear localization by suppressing the activity of CaM-kinase2 (CAMK2) via the SRC/YES kinase pathway [8].

The present study demonstrates that MIB2-mediated YAP/TAZ degradation plays a crucial role in the tumor-suppressive function of FAT1. We found that re-expressing FAT1 in FAT1-deficient tumors reduces their tumorigenic potential, an effect that depends on the presence of MIB2. In tumor cells, FAT1 interacts with the E3 ligase MIB2 to facilitate the ubiquitin-dependent degradation of YAP/TAZ. Loss of either FAT1 or MIB2 significantly elevates YAP/TAZ protein levels, enhances their transcriptional activity, and promotes tumor cell proliferation in both in vitro and in vivo models. These findings establish a critical link between FAT1, MIB2, and the regulation of YAP/TAZ in tumor progression.

## Results

### Loss of FAT1 expression results in increased YAP and TAZ protein levels

**Fig 1:**
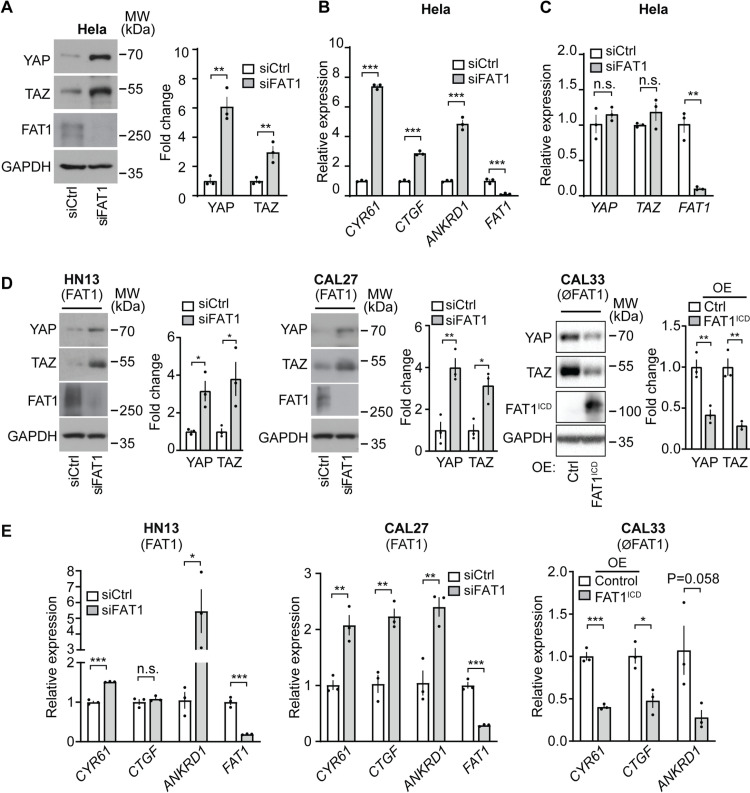
Loss of FAT1 expression results in increased YAP/TAZ protein levels. (A) YAP/TAZ protein levels in Hela cells transfected with control siRNA or siRNA directed against *FAT1*. Shown is a representative experiment out of 3 independently performed experiments and the statistical analysis (n = 3). (B) Expression of the indicated YAP/TAZ target genes in Hela cells after transfection with control siRNA or siRNA directed against *FAT1* (n = 3). (C) YAP, TAZ, and FAT1 mRNA levels in Hela cells following transfection with either control siRNA or FAT1-targeted siRNA. (n = 3 independent experiments). (D, E) YAP/TAZ protein levels (D) and expression of YAP/TAZ target genes (E) in the FAT1-expressing HNSCCs HN13 and CAL27 as well as in FAT1-deficient CAL33 cells, which were either transfected with control siRNA or siRNA directed against FAT1 (HN13 and CAL27) or which were infected with a lentivirus delivering a DNA construct encoding the intracellular domain of FAT1 linked to the transmembrane domain (TMD) of the IL-2 receptor (FAT1^ICD^) or encoding just the TMD of the IL-2 receptor (control) (CAL33). Shown are Western blots representative of 3 independently performed experiments and the statistical analysis (D) or the analysis of 3 independently performed experiments (E). OE, over expression. Data are presented as mean ± SEM. *, P ≤ 0.05; **, P ≤ 0.01; ***, P ≤ 0.001; n.s., not significant (two-tailed unpaired *t*-test).

When studying the function of FAT1 in the cervical cancer cell line Hela, we noticed an increase in YAP and TAZ protein levels after knock-down of FAT1 (Fig 1A). The suppression of FAT1 led to an upregulation of YAP/TAZ target gene expression (Fig 1B) without affecting YAP and TAZ mRNA levels (Fig 1C). This suggests that the elevated YAP/TAZ protein levels observed after FAT1 knockdown were not due to increased transcription or enhanced mRNA stability of YAP and TAZ. To test whether FAT1-mediated regulation of YAP/TAZ protein levels is a more general mechanism, we analyzed the breast cancer cell line MDA-MB-231 and the glioblastoma cell line U-87 transfected with control siRNA and siRNA directed against *FAT1*. In all studied cell lines, suppression of *FAT1* expression resulted in increased protein levels of YAP and TAZ (S1A and B Fig) as well as in increased expression of YAP/TAZ target genes (S1C and D Fig).

We also analyzed YAP/TAZ protein levels in cells derived from head and neck squamous cell carcinoma (HNSCC). We selected HNSCCs which either expressed *FAT1* or which had lost *FAT1* expression [2,6] but lacked mutations in genes encoding proteins operating downstream of FAT1 such as MIB2 and YAP/TAZ [6] (S2 Fig). Suppression of *FAT1* expression in the tumorigenic HNSCCs HN13 and CAL27, which express FAT1 (S2 Fig), resulted in increased protein levels of YAP and TAZ and increased expression of YAP/TAZ target genes (Fig 1D and E). In contrast, the HNSCC CAL33, which lacks full length *FAT1* expression due to a mutation in exon 18 resulting in a premature stop codon [6], showed increased YAP/TAZ protein levels (S2 Fig). However, expression of the intracellular domain of FAT1 fused to the transmembrane domain of the IL-2 receptor (FAT1^ICD^) in CAL33 cells rescued this phenotype and led to reduced YAP/TAZ protein levels and reduced expression of YAP/TAZ target genes (Fig 1D and E).

### FAT1 controls degradation of YAP and TAZ protein

**Fig 2:**
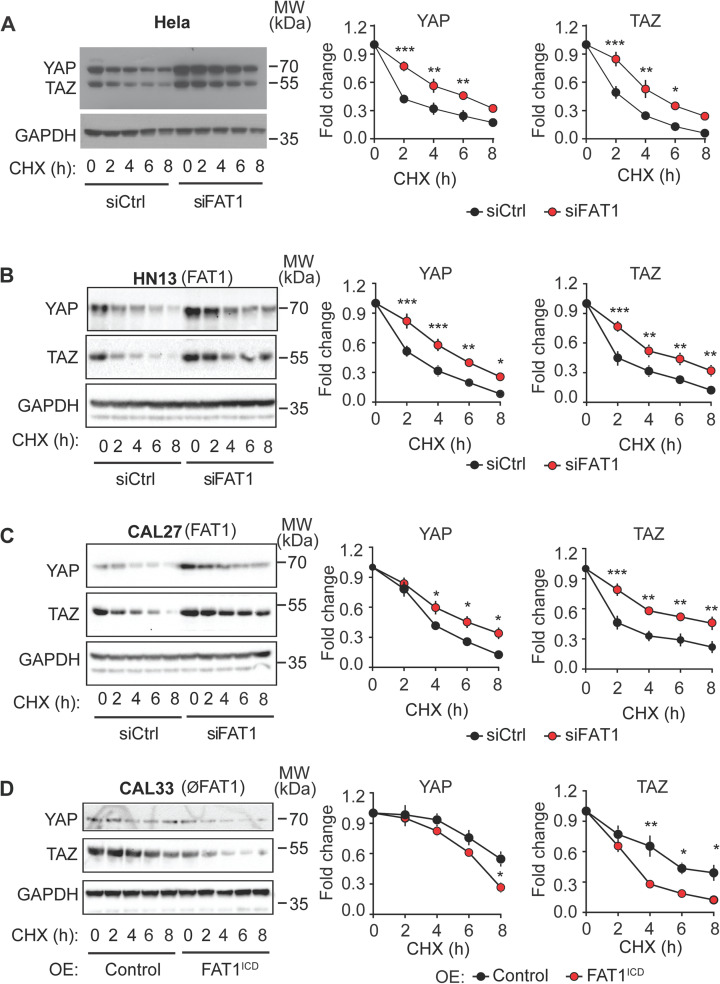
FAT1 controls degradation of YAP and TAZ proteins. (A-D) YAP and TAZ protein degradation in Hela cells (A) and in the HNSCC HN13, CAL27 and CAL33 (B-D) transfected with control siRNA or siRNA directed against *FAT1* (A-C) or expressing the FLAG-tagged intracellular domain of FAT1 linked to the TMD of the IL-2 receptor (FAT1^ICD^) or just the TMD of the IL-2 receptor (control) (D) was analyzed after incubation of cells with 50 µg/ml cycloheximide (CHX) for the indicated time periods. Shown are the YAP and TAZ protein levels as determined by immunoblotting. Diagrams show the statistical evaluation (n = 3 independent experiments; data are normalized to the basal level of YAP and TAZ at time point zero). Data are presented as mean ± SEM. *, P ≤ 0.05; **, P ≤ 0.01; ***, P ≤ 0.001 (two-way ANOVA and Bonferroni’s post-hoc test).

To investigate whether FAT1 loss impacts YAP/TAZ protein degradation in tumor cells, we examined YAP and TAZ levels by treatment of protein synthesis inhibitor cycloheximide. In Hela, MDA-MB-231, U-87, and the HNSCC cell lines HN13 and CAL27, inhibiting protein synthesis led to a rapid decline in YAP and TAZ protein levels, dropping to below half within 2–4 hours (Fig 2A–C; S3A and B Fig). However, following FAT1 suppression, basal YAP and TAZ levels were significantly elevated, and the inhibitory effect of cycloheximide on YAP/TAZ degradation was substantially weakened (Fig 2A–C; S3A and B Fig). These results indicate that FAT1 loss impairs YAP/TAZ protein degradation.

Consistent with a role of FAT1 in promoting YAP/TAZ protein degradation, YAP/TAZ protein levels were less affected by protein synthesis inhibition in the FAT1-deficient HNSCC CAL33 (Fig 2D), whereas expression of FAT1^ICD^ in CAL33 cells reduced YAP/TAZ protein levels and led to further increased protein degradation after cycloheximide treatment (Fig 2D).

### The intracellular domain of FAT1 interacts with E3 ligase MIB2

**Fig 3:**
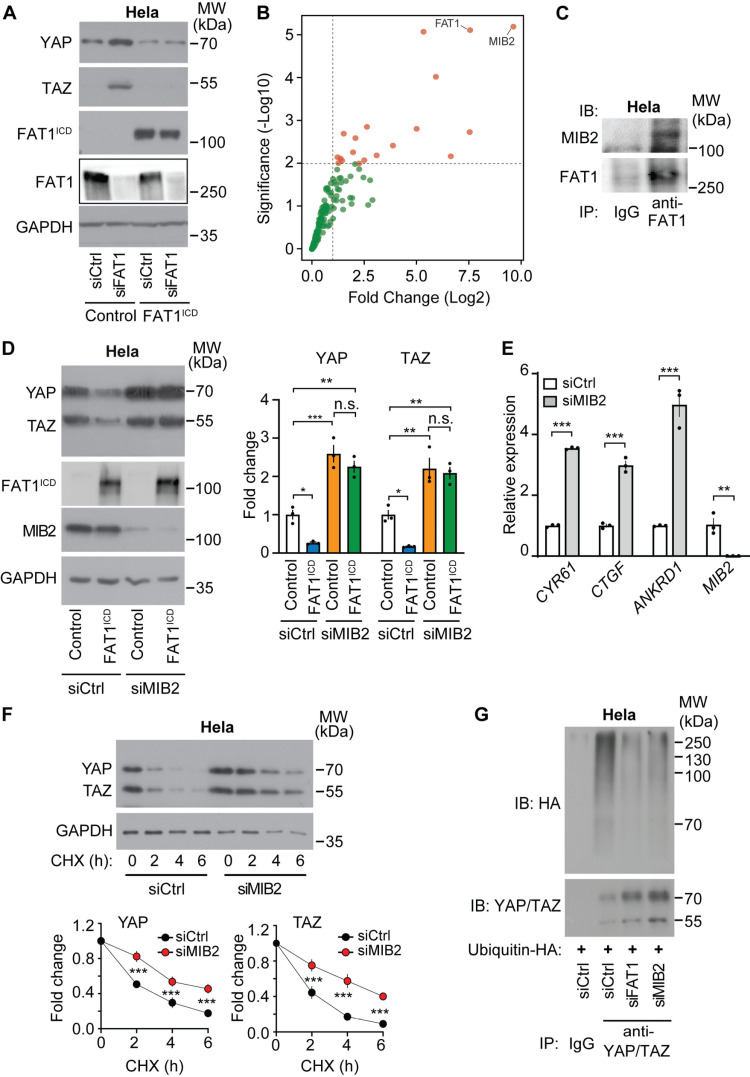
FAT1 interacts with the E3 ligase MIB2. (A-B) Hela cells were transfected with a (control) vector or a plasmid encoding FAT1^ICD^ to generate stably expressing cells. Hela cells expressing either control or FAT1^ICD^ were transfected with control siRNA or siRNA targeting endogenous FAT1, followed by immunoblotting to assess YAP and TAZ protein levels. (A). Shown is a representative of 3 independently performed experiments. The FAT1^ICD^-FLAG protein was precipitated from lysates of stably transduced Hela cells using an anti-FLAG antibody. The immunoprecipitated proteins were subsequently analyzed using mass spectrometry (B). The volcano plot illustrates proteins significantly enriched in the FAT1^ICD^ interactome. (C) Hela cells were lysed, and endogenous FAT1 was immunoprecipitated. Immunoprecipitates were then analyzed by immunoblotting with antibodies against MIB2. Control IgG was used for immunoprecipitation as a negative control. The data presented represent one of three independently conducted experiments. (D, E) Hela cells (transfected without or with FAT1^ICD^) were in addition transfected with control siRNA or siRNA directed against *MIB2* as indicated. Thereafter, YAP/TAZ protein levels were analyzed by immunoblotting (D; shown is a representative of 3 independently performed immunoblot experiments and the statistical evaluation), or cells were analyzed for expression of YAP/TAZ target genes (E, n = 3 independently performed experiments). (F) Hela cells were transfected with control siRNA or siRNA directed against *MIB2*. YAP and TAZ protein degradation was determined by incubation of cells with cycloheximide (CHX) for the indicated time periods and subsequent analysis of YAP/TAZ protein levels by immunoblotting. Shown is 1 out of 3 independently performed experiments with the statistical analysis shown on the right. (G) Hela cells were transfected with a plasmid encoding HA-tagged ubiquitin as well as with control siRNA or siRNA directed against *FAT1* or *MIB2* as indicated. Thereafter, cells were lysed, and YAP and TAZ were immunoprecipitated. Shown is the analysis of the immunoprecipitates by immunoblotting with antibodies against HA-tagged ubiquitin and YAP/TAZ. Shown is one representative of three independently performed experiments. Shown are mean values ± SEM. *, P ≤ 0.05; **, P ≤ 0.01; ***, P ≤ 0.001; n.s., not significant (one-way ANOVA plus Tukey’s post-hoc test (D), two-tailed unpaired t-test (E), two-way ANOVA and Bonferroni’s post-hoc test (F)).

As shown in Fig 3A, the increase in YAP and TAZ protein levels induced by FAT1 knock-down in Hela cells was fully normalized by reexpression of the FLAG-tagged intracellular domain of FAT1. Since we recently described that in endothelial cells the intracellular domain of FAT1 interacts with the E3-ligase MIB2 to control YAP/TAZ protein degradation [26], we tested whether this interaction also occurs in tumor cells. We therefore expressed FLAG-tagged FAT1^ICD^ in Hela cells, immunoprecipitated it using an anti-FLAG antibody from cell lysates and subjected the precipitates to mass spectrometry-based analysis of the interactome. The analyses identified several highly enriched proteins in the anti-FLAG-IL-2R-FAT1-ICD precipitates. Among these, the E3 ligase MIB2 stood out as the most prominent protein compared to the controls (Fig 3B, S1 Table).

MIB2 was also found to coprecipitate with endogenous wild-type FAT1 in Hela cells (Fig 3C), and knockdown of MIB2 in Hela cells basically recapitulated the effect of a FAT1 knockdown and resulted in increased YAP/TAZ protein levels, in elevated expression of YAP/TAZ target genes and in reduced YAP/TAZ protein degradation (Fig 3D–F). Overexpression of the intracellular domain of FAT1 in Hela cells reduced YAP/TAZ levels in control cells (Fig 3D) but had no effect after knock-down of MIB2 (Fig 3D), indicating that MIB2 indeed operates downstream of FAT1. We further investigated whether the interaction between MIB2 and FAT1 impacts YAP/TAZ ubiquitination by immunoprecipitating YAP and TAZ from Hela cells. Under normal conditions, YAP/TAZ ubiquitination was readily detectable. However, silencing FAT1 or MIB2 expression with specific siRNAs led to a marked reduction in ubiquitination levels (Fig 3G). These findings collectively demonstrate that FAT1 cooperates with MIB2 to facilitate the ubiquitination of YAP/TAZ in Hela cells.

### MIB2 facilitates FAT1 dependent degradation of YAP/TAZ proteins

**Fig 4:**
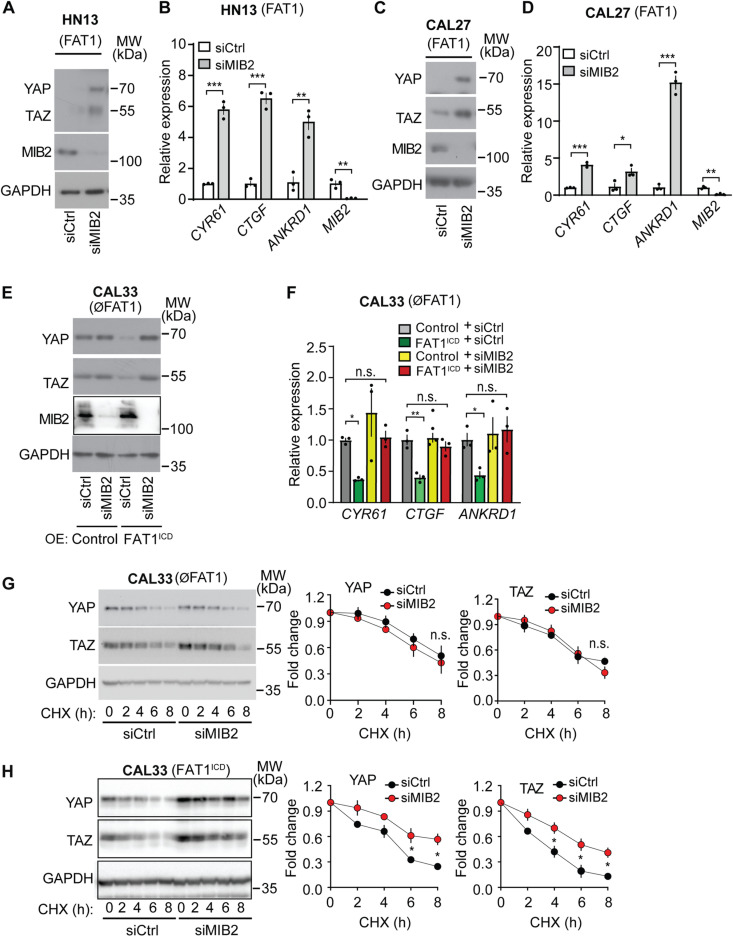
Regulation of YAP/TAZ through MIB2 in HNSCCs. (A-H) HNSCCs expressing *FAT1* (HN13 and CAL27) and the FAT1-deficient HNSCC CAL33 expressing a control or FAT1^ICD^ were transfected with control siRNA or siRNA directed against *MIB2*. Untreated cells (A-F) or cells incubated with 50 µg/ml CHX for the indicated time periods (G, H) were then lysed, and YAP and TAZ protein levels were determined by immunoblotting (A, C, E, G, H), or expression of YAP/TAZ target genes was analyzed by RT-qPCR (B, D, F) (n = 3). The presented immunoblots are representative examples of 3 independently performed experiments. Diagrams in G and H show the densitometric evaluation of blots (n = 3). Shown are mean values ± SEM. *, P ≤ 0.05; **, P ≤ 0.01; ***, P ≤ 0.001; n.s., not significant (two-tailed unpaired t-test (B and D)), two-way ANOVA and Bonferroni’s post-hoc test (G, H); one-way ANOVA plus Tukey’s post-hoc test (F).

To investigate the role of MIB2, we performed siRNA-mediated knockdown in multiple tumor cell lines. Reducing MIB2 expression resulted in elevated YAP and TAZ protein levels in MDA-MB-231 and U-87 cells (S4A and C Fig). This upregulation was further associated with enhanced expression of YAP/TAZ target genes (S4 B and D Fig). Similarly, in the HNSCC HN13 and CAL27, which express *FAT1*, knock-down of MIB2 led to in increased YAP/TAZ levels (Fig 4A and C), increased expression of YAP/TAZ target genes (Fig 4B and D) and reduced YAP/TAZ protein degradation (S5A and B Fig). In the HNSCC CAL33, which lacks FAT1, knock-down of MIB2 had no effect on YAP/TAZ protein levels (Fig 4E). However, when the intracellular domain of FAT1 was expressed in CAL33 cells, protein levels of YAP and TAZ as well as expression of YAP/TAZ target genes was reduced, and knockdown of MIB2 reverted this effect, resulting in YAP/TAZ protein levels and YAP/TAZ target gene expression similar to those found in control CAL33 cells (Fig 4E and F). Similarly, knockdown of MIB2 has no effect on YAP/TAZ protein degradation in the parental CAL33 cells. However, after expression of FAT1 intracellular domain, YAP/TAZ degradation was increased, and this effect was gone after knockdown of MIB2 (Fig 4G and H).

**Fig 5:**
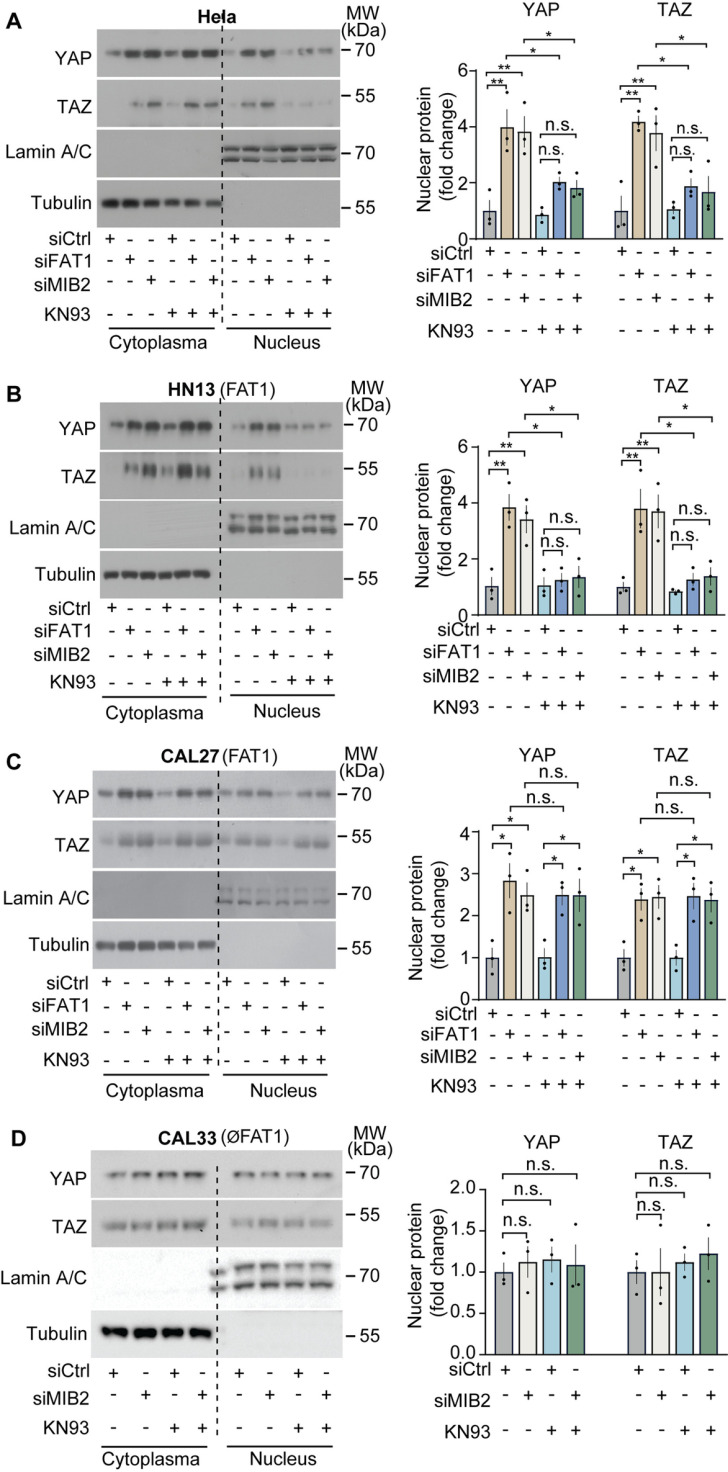
Role of MIB2 and CAMK2 in FAT1-depndent regulation of YAP/TAZ protein levels and YAP/TAZ nuclear localization. (A-D) Hela cells (A) or HNSCC cells expressing *FAT1* (HN13 and CAL27; B, C) or lacking FAT1 (CAL33; D) were transfected with control siRNA or siRNA directed against *FAT1* or *MIB2* and were then treated in the absence (-) or presence (+) of 10 µM of the CAMK2 inhibitor KN93. Thereafter, cells were lysed, and cytoplasmic and nuclear factions were analyzed by immunoblotting using antibodies against YAP ad TAZ. Lamin A/C and tubulin served as controls for proper fractionation. Shown is a representative experiment of 3 independently performed experiments and the statistical analysis. Shown are mean values ± SEM. *, P ≤ 0.05; **, P ≤ 0.01; n.s., not significant (one-way ANOVA plus Tukey’s post-hoc test).

Recent evidence suggests that CAMK2 mediates the nuclear translocation of YAP/TAZ in the absence of FAT1 [8]. We therefore studied the effect of pharmacological inhibition of CAMK2 with KN93 on the YAP/TAZ protein level in the cytosol as well as in the nucleus of Hela cells and of the FAT1-expressing HNSCC HN13 and CAL27 without and with knock-down of FAT1 or MIB2. We found that both suppression of *FAT1* and *MIB2* expression increased total and nuclear protein levels of YAP and TAZ (Fig 5A–C). Interestingly, inhibition of CAMK2 with KN93 blocked nuclear increase of YAP and TAZ in Hela cells and in HN13 cells but not in CAL27 cells (Fig 5A–C). In CAL33 cells, which lack FAT1 expression, knock-down of MIB2 had no effect on nuclear localization of YAP and TAZ, and inhibition of CAMK2 did not change nuclear YAP and TAZ localization (Fig 5D). These data indicate that MIB2 inhibits nuclear localization of YAP and TAZ by promoting YAP and TAZ degradation and that in some but not all cells CAMK2 promotes nuclear localization of YAP and TAZ.

### Loss of MIB2 mimics effect of loss of tumor suppressor FAT1 on tumor cell growth *in vitro*

**Fig 6:**
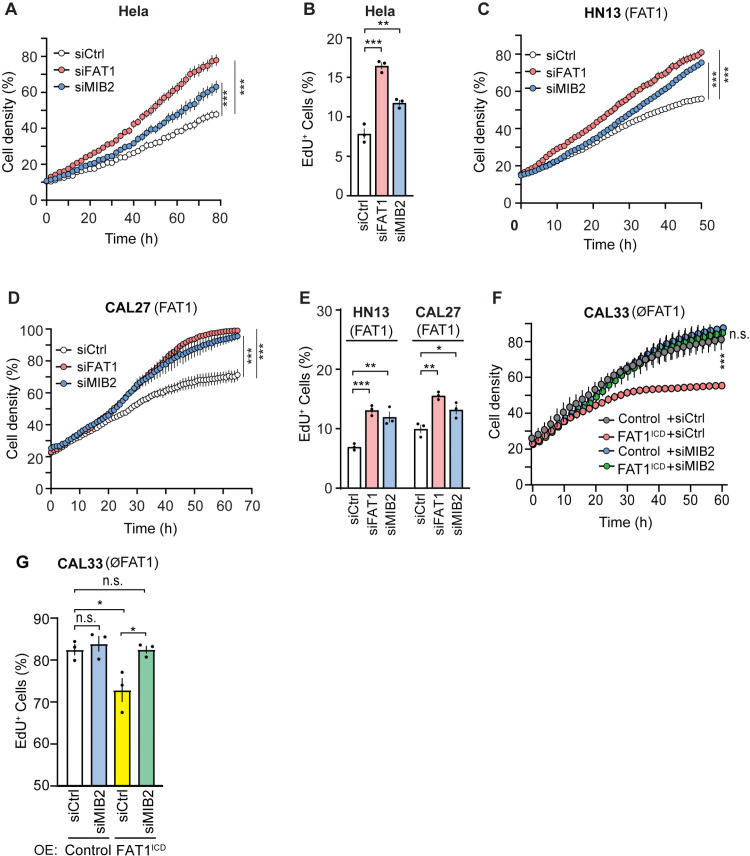
Role of MIB2 in tumor cell growth *in vitro.* (A-G) Hela cells (A,B) and HNSCCs (C-G) transfected with control siRNA or siRNA directed against *FAT1* (A-E) or *MIB2* (A-G) or transduced without or with FAT1^ICD^ (F, G) were analyzed for cell proliferation using an IncuCyteZoom live cell analysis system (n = 4 in A, C, D and n = 3 in F). In parallel, EdU incorporation was determined by flow cytometry (B, E, G; n = 3). Shown are mean values ± S.E.M; *, P ≤ 0.05; **, P ≤ 0.01; ***, P ≤ 0.001; n.s., not significant (two-way ANOVA and Bonferroni’s post-hoc test (A, C, D, F) and one-way ANOVA and Tukey’s post-hoc test (B, E, G)).

To investigate whether suppression of *MIB2* expression mimics the effect of a loss of *FAT1* on tumor cell growth, we suppressed expression of *FAT1* or *MIB2* using siRNA in Hela cells as well as in HNSCCs lacking or expressing FAT1 followed by evaluation of cell growth and EdU incorporation. Proliferation as well as EdU incorporation of Hela cells with suppressed expression of *FAT1* or *MIB2* was increased (Fig 6A and B). Similarly, proliferation and EdU incorporation of the tumorigenic HNSCC HN13 and CAL27, which express *FAT1*, increased after knock-down of FAT1 or MIB2 (Fig 6C–E). In contrast, knockdown of MIB2 had no effect on the proliferation and EdU incorporation of the HNSCC CAL33, which lacks FAT1 (Fig 6F and G), but expression of the intracellular part of FAT1 led to reduced proliferation, an effect which could be blocked by knock-down of MIB2 (Fig 6F and G). The increased proliferation and EdU incorporation after knock down of FAT1 and MIB2 in CAL27 cells was rescued by additional knock down of YAP/TAZ (S6A, B Fig). At the same time, the decreased proliferation and EdU incorporation after FAT1 overexpression in CAL33 was reverted by overexpression of YAP (S6C and D Fig). These data indicate that loss of MIB2-mediated YAP/TAZ protein degradation and subsequent increase in YAP/TAZ protein levels is responsible for the enhanced tumor cell proliferation in the absence of FAT1.

### FAT1 and MIB2 control tumor growth *in vivo*

**Fig 7:**
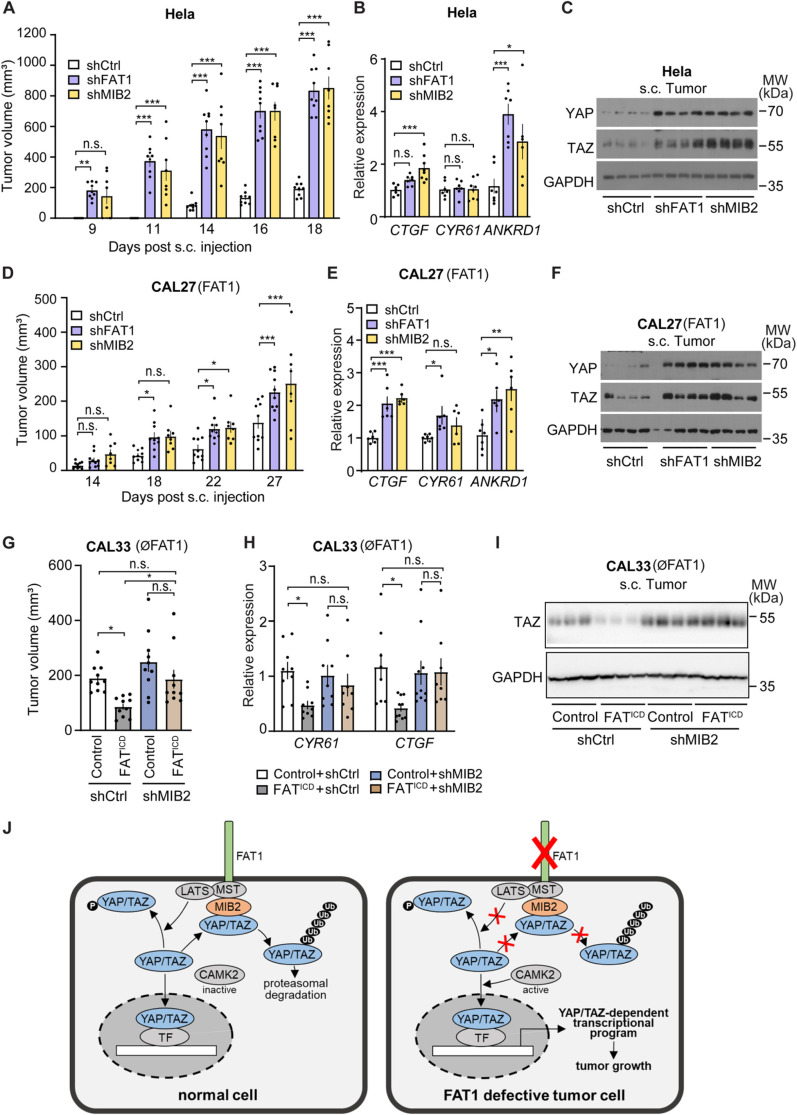
FAT1 and MIB2 control tumor growth *in vivo.* (A-F) NOG mice were injected subcutaneously with Hela cells (A-C) or with the HNSCC CAL27 (D-F) transduced with control shRNA or shRNA directed against *FAT1* or *MIB2.* Tumor volume was measured every 2-4 days (n = 9 mice (Hela, control and shFAT1); n = 10 (CAL27, control and shFAT1) and n = 8 mice (Hela and CAL27, shMIB2)). (B, E) Expression of YAP/TAZ target genes in the tumor tissue was analyzed by qPCR (n = 7 mice (Hela, control and shFAT1; CAL27, control) and n = 6-8 mice (Hela and CAL27, shMIB2; CAL27, shFAT1)). (C, F) YAP and TAZ protein levels in tumors were analyzed by Western blotting. Shown are the results of 4 representative mice per group. (G-I) Nude mice were injected subcutaneously with the HNSCC CAL33 expressing control or the intracellular domain of FAT1 fused to the IL-2 receptor transmembrane domain (FAT1^ICD^) and transduced with control shRNA or shRNA directed against *MIB2*. The tumor volume was determined after 2 weeks (n = 10 mice per group) (G). (H, I) Expression of YAP/TAZ target genes in the tumor tissue was analyzed by qPCR (n = 8-10) (H), and YAP and TAZ protein levels in tumors were analyzed by Western blotting (I; shown are the results of 3 representative mice per group). (J) Schematic representation showing how FAT1 negatively controls tumor cell proliferation through MIB2-mediated degradation of YAP/TAZ. TF, transcription factor; Ub, ubiquitin; MST, mammalian sterile 20-like kinase; LATS, large tumor suppressor kinase. Shown are mean values ± S.E.M; *, P ≤ 0.05; **, P ≤ 0.01; ***, P ≤ 0.001; n.s., not significant (two-way ANOVA and Bonferroni’s post-hoc test (A, D), one-way ANOVA and Tukey’s post-hoc test (B, E, G) and Kruskal-Wallis test (H)).

We then suppressed expression of *FAT1* or *MIB2* using lentiviral transfer of shRNA directed against *FAT1* or *MIB2* in Hela cells and in the FAT1-expressing HNSCC CAL27 (S7A and B Fig) and analyzed the growth of tumor cells *in vivo* after subcutaneous injection. While control Hela cells injected into NOG mice lead to tumor formation after 14 days, Hela cells with knock-down of FAT1 or MIB2 developed visible tumors as early as 9 days after injection (Fig 7A). The increased tumor growth of Hela cells lacking FAT1 and MIB2 was accompanied by increased expression of YAP/TAZ target genes and increased levels of YAP/TAZ protein in the tumors (Fig 7B and C). Similar to Hela tumor cells with suppressed expression of FAT1 and MIB2, we also saw increased tumor growth in the HNSCC CAL27 with suppressed FAT1 and MIB2 expression after subcutaneous injection of cells into NOG mice (Fig 7D). The increase in tumor growth in the absence of FAT1 and MIB2 was again accompanied by increased expression of YAP/TAZ target genes as well as of YAP/TAZ protein (Fig 7E and F).

Finally, we compared *in vivo* tumor growth of the HNSCC CAL33 which lacks FAT1 with the CAL33 expressing the intracellular domain of FAT1 (S7C Fig.). We found that FAT1^ICD^-expressing CAL33 cells resulted in tumors of smaller size compared to the parental line (Fig 7G). When the same experiment was performed in CAL33 cells, in which MIB2 expression was suppressed, expression of FAT1^ICD^ had no effect any more (Fig 7G). Target gene expression and YAP/TAZ protein levels were reduced in FAT1^ICD^ expressing CAL33 cells, and this effect was reverted by knock-down of MIB2 (Fig 7H and I). Thus, also under *in vivo* conditions, was the ability of FAT1 to increase YAP/TAZ protein degradation and to function as a tumor suppressor dependent on MIB2.

## Discussion

Loss-of-function mutations in the tumor suppressor gene FAT1 have been shown to drive tumor progression and enhance YAP/TAZ signaling. [2,3]. However, the mechanism by which FAT1 regulates YAP/TAZ has remained somewhat unclear. In recent years, two mechanisms operating in tumor cells have been described [2,8]. FAT1 can assemble the Hippo signaling complex consisting of various protein kinases including MST and LATS, which results in YAP inactivation [2]. In addition, FAT1 has also been reported to impede YAP nuclear translocation by inhibiting CAMK2 through only partially understood mechanisms [8]. In the absence of FAT1, CAMK2, through CD44 and SRC/YES, promotes nuclear translocation of YAP and TAZ. Our results show that FAT1 plays a role in promoting YAP/TAZ protein degradation by interacting with the E3 ligase MIB2, which drives their ubiquitination. Disruption of this mechanism results in elevated YAP/TAZ protein levels, increased expression of their target genes, and enhanced tumor progression. (Fig 7J). Our data also explain previous observations of increase in YAP/TAZ protein levels after loss of FAT1 in tumor cells [8,9].

How do the mechanisms linking FAT1 to decreased YAP/TAZ activity function together? Mutagenesis of the FAT1 intracellular domain had shown that the most upstream kinase of the Hippo pathway, MST1/2, primarily interacts with a region comprising the first 58 amino acids of the intracellular domain close to the plasma membrane, whereas a second, less strong interaction was observed with the last 186 amino acids in the very C-terminal region of FAT1 [2]. Interestingly, the interaction of MIB2 with the intracellular domain of FAT1 was mapped to a region between amino acids 58 and 200 of the intracellular domain of FAT1 [26]. Thus, the binding regions for MST1/2 and MIB2 are mutually exclusive, suggesting that FAT1 can interact with both MST1/2 and MIB2 and thereby regulate YAP/TAZ through the Hippo pathway as well as through MIB2-mediated degradation. How FAT1 inhibits CAMK2 [8], is not known. While we found in all tested tumor cells that YAP/TAZ protein levels were regulated by FAT1 in a MIB2-dependent manner, not all cells showed CAMK2-dependent nuclear translocation. This shows that MIB2 and CAMK2-dependent regulation of YAP/TAZ by FAT1 can function in parallel but also independently of each other. However, in all tumor cells studied by us, loss of MIB2-mediated YAP/TAZ degradation appeared to be a requirement for increased tumor progression in the absence of FAT1 function.

YAP and TAZ have been described to be ubiquitinated for subsequent proteasomal degradation in different ways. The Hippo pathway eventually leads to phosphorylation of YAP and TAZ at serine 381 and 311, which leads to their ubiquitination and degradation by the SCFβ-TrCP E3 ubiquitin ligase [27,28]. In hepatocellular carcinoma cells, YAP has been shown to undergo ubiquitination mediated by the E3 ligase F-box and WD repeat domain-containing 7 (Fbxw7), with an inverse relationship observed between Fbxw7 expression and YAP protein levels [29]. The E3 ligase RNF187 facilitates YAP ubiquitination in triple-negative breast cancer cells, where decreased RNF187 expression is linked to increased YAP protein levels [30]. Our findings expand the list of E3 ubiquitin ligases involved in regulating YAP/TAZ protein degradation in the context of tumor growth to MIB2 and show that in FAT1-expressing tumor cells MIB2 is centrally involved in controlling YAP/TAZ protein levels and YAP/TAZ downstream signaling and that the FAT1/MIB2 pathway functions as a critical tumor suppressor.

MIB2 is an E3 ligase, which has recently been shown to mediate ubiquitination of PD-L1 in murine melanoma B16 cells and colorectal carcinoma MC38 cells, and to thereby facilitate the transport of PD-L1 from the trans-Golgi network to the plasma membrane to prevent T-cell-mediated tumor cells death [31]. To what degree MIB2-mediated effects on PD-L1 signaling or transport contribute to the effects of FAT1 is unknown. MIB2 has also been shown to promote Notch signaling by ubiquitination of Notch ligands, such as Delta and Jagged [32–34]. Given that Notch can be either oncogenic or tumor-suppressive depending on the cellular context in various tumors including HNSCC [35–39], it is conceivable that loss of FAT1 affects Notch signaling in tumor cells through altered localization of MIB2.

Extensive evidence has been provided that YAP and TAZ are important drivers of solid tumor growth, epithelial-to-mesenchymal transition (EMT), metastasis and resistance to therapy [12,13,15,17,40]. Recent data have not only shown that loss of FAT1 expression leads to increased YAP/TAZ activity and expression of YAP/TAZ target genes but also that increased YAP/TAZ activity is responsible for increased tumor growth, EMT, metastasis and resistance to therapy in tumors lacking FAT1 [2,3,8]. Thus, suppression of YAP/TAZ activity and YAP/TAZ target gene activity by FAT1 is critical for its role as a tumor suppressor. The fact that loss of MIB2-mediated increase in YAP/TAZ protein degradation had the same effect on YAP/TAZ protein levels and YAP/TAZ target gene activation as the loss of FAT1 indicates that loss of MIB2-mediated increase in YAP/TAZ protein degradation is a critical mechanism, by which mutations in *FAT1* promote tumorigenesis and tumor progression. This is supported by our *in vivo* data demonstrating that loss of FAT1 as well as of MIB2 increased YAP/TAZ protein levels and tumor growth of Hela cells as well as of HNSCCs and that FAT1-mediated reduction in tumor progression depended on MIB2.

## Methods

### Reagents

MG132 (catalog #M8699), N-Ethylmaleimide (catalog #E3876), cycloheximide (catalog #239763-M), puromycin (catalog #P8833), and KN93 (catalog #K1385) were purchased from Sigma-Aldrich. EdU (catalog #40540) was ordered from Lumiprobe GmbH. Blasticidin was ordered from MP Biomedicals (catalog #150477) Fetal bovine serum (FBS, catalog # 10270106) and Click-iT^TM^ EdU Alexa Fluor^TM^ 647 flow cytometry assay kit (catalog #C10424) were from Thermo Fisher Scientific. Dulbecco’s Modified Eagle Medium (DMEM, catalog #10938−025), sodium pyruvate (catalog #11360−039), penicillin and streptomycin (catalog #15140−122) and L-glutamine (catalog #25030−024) were obtained from Gibco. Antibodies directed against FAT1 (ab190242) were from Abcam. Antibodies directed against HA (catalog #H6533) and Flag M2 (catalog #A8592), α-Tubulin (catalog #T9026) were from Sigma-Aldrich. Antibodies directed against YAP/TAZ (catalog #8418), YAP (catalog #4912), Lamin A/C (catalog #2032), GAPDH (catalog #2118), CaMKII (catalog #4436) and ubiquitin (catalog #3936) were obtained from Cell Signaling. The anti-MIB2 antibody (catalog #A301-414A) was from Bethyl Laboratories.

### Cell culture

Hela, MDA-MB-231 and U87 were from ATCC. The HNSCCs HN13 and CAL27 were described before [2,41]. CAL33 was purchased from the Deutsche Sammlung von Mikroorganismen und Zellkulturen (DSMZ). All cells were cultured in DMEM medium supplemented with 10% FBS, 1% penicillin/streptomycin, 1 mM sodium pyruvate and 2 mM L-glutamine at 37 °C and under a humidified atmosphere of 5% CO_2_ and were tested negative for mycoplasma contamination before experiments.

### siRNA-mediated knockdown

Cells at 50−70% confluence were transfected with siRNA using Opti-MEM and Lipofectamine RNAiMAX (Invitrogen) as described previously [42]. Control siRNA and siRNAs directed against *FAT1, MIB2, YAP* and *TAZ* were from Sigma. The targeted sequences of those siRNAs were as follows: *FAT1* (SASI_Hs01_00232012), 5-CATCGAACAGGCCAATGAA-3; *MIB2* (SASI_Hs01_00031060), 5-GTGTGTGCCTGGACTACGA-3; *YAP* (SASI_Hs01_00182402), *WWTR1* (SASI_Hs01_00124477).

### Western blotting

Cells were lysed in 1 x Laemmli buffer directly containing 50 mM Tris-HCl (pH 6.8), 2.5 mM EDTA, 1% (w/v) SDS, 5% glycerol, 1% β-mercaptoethanol, 50 µg/ml bromophenol blue. Total cell lysates were subjected to sodium dodecyl sulfate-polyacrylamide gel electrophoresis (SDS-PAGE). Protein was then transferred to nitrocellulose membranes, followed by overnight incubation with primary antibodies. Membranes were incubated with horseradish peroxidase-conjugated (HPC) secondary antibodies (Cell Signaling Technology) for 2 h at room temperature and were developed using the ECL detection system (Thermo Scientific Pierce, Life Technologies). Protein band intensities were analyzed by ImageJ software (NIH). In some cases, cells treated with KN93 (10 µM) or control vehicle for 36 hours were fractionated to distinguish nuclear and cytoplasmic proteins prior to Western blot analysis. Cell fractionation was performed using the NE-PER extraction reagent (ThermoFisher Scientific, catalog #78833) according to the manufacturer’s instructions.

### Immunoprecipitation

For immunoprecipitation of endogenous FAT1, Hela cells were lysed in immunoprecipitation (IP) buffer (150 mM NaCl, 25 mM Tris-HCl (pH 7.4), 1 mM EDTA, 1% NP-40, 5% glycerol) supplemented with protease inhibitor cocktail (catalog #4693159001, Roche) on ice. Samples were centrifuged at 30,000 x g for 15 min. Supernatant was incubated with 2 µg of the anti-FAT1 antibody overnight at 4 °C under gentle rotation. Thereafter, A/G-Sepharose beads (catalog #sc-2003, Santa Cruz) pre-equilibrated in IP buffer were added, and the sample was incubated for 1 h at 4 °C. A/G-Sepharose beads were then collected by centrifugation at 3.500 x g for 1 min, and proteins were eluted with Laemmli buffer after being washed 3 times. Samples were subjected to SDS-PAGE.

### RNA isolation and quantitative RT-PCR

Total RNA was isolated from cells or tissues using Quick-RNA ^TM^ MicroPrep Kit (Zymo Research). Quality control of RNAs were done with Nanodrop ND-100 Spectrophotometer. RNA was reverse-transcribed using ProtoScript II First Strand cDNA Synthesis Kit (New England Biolabs) according to manufacturer’s instructions. Quantitative RT-PCR was done with TaqMan Probe and primers were designed with the Roche’s online tool and a Universal Probe Library assay (Roche). Relative expression levels were calculated after normalizing with GAPDH expression levels. The following primer sequences were used: *FAT1* (human) #81: 5’-GAGCTGCCGAGGACTTTAGA-3’, 5’-TGCTTAACTGTCGGGAATCA-3’; MIB2 (human) #11: 5’-TACAAGGACCACCTCCCAAG-3’, 5’-TGTCCAGCAGACACTTGACC-3’; *CYR61* (human) #7: 5’-TCCAGGGCACACCTAGACA-3’, 5’-GCCCATTTTCTCCATGATTC-3’; *ANKRD1* (human) #61: 5’-GATCGAATTCCGTGATATGCT-3’, 5’-AAACATCCAGGTTTCCTCCA-3’; *CTGF* (human) #85: 5’-GCCTCCTGCAGGCTAGAGA-3’, 5’-GATGCACTTTTTGCCCTTCT-3’; *YAP* (human) #47: 5’-ATCCCAGCACAGCAAATTCT-3’, 5’-TGGATTTTGAGTCCCACCAT-3’; *TAZ* (human) #7: 5’-ATTCGAATGCGCCAAGAG-3’, 5’-AACTGGGGCAAGAGTCTCAG-3’; *GAPDH* (human) #82: 5’-GCATCCTGGGCTACACTGA-3’, 5’-CCAGCGTCAAAGGTGGAG −3’.

### Cycloheximide chase experiment

Cells were transfected with the indicated siRNAs or infected with control vector or vector expressing FAT1^ICD^, following the protocol mentioned above. 48 h after first transfection, cells were treated with 50 µg/ml cycloheximide for the indicated time periods and then collected in 1 x Laemmli blue buffer for further analysis by immunoblotting.

### Determination of ubiquitination

Hela cells were transfected with siRNAs against the indicated RNAs following the protocol described above. 8–12 h after the second transfection, cells were treated with 20 µM MG132 for 16 h. Cells were lysed in lysis buffer containing 150 mM NaCl, 10 mM Tris-HCl (pH 8.0), 2% SDS, 2 mM sodium orthovanadate, 50 mM sodium fluoride, 20 nM NEM as well as protease inhibitor tablet (catalog #4693159001, Roche) for 30 min on ice. Whole cell lysates were boiled for 10 min and then diluted to 10 x volume with dilution buffer (150 mM NaCl, 10 mM Tris-HCl (pH 8.0), 2 mM EDTA, 1% Triton X-100). Samples were centrifuged at 16,000 x g for 15 min, and the supernatants were incubated with YAP/TAZ antibody and protein-A- or G-agarose for 4–5 h at 4 °C. Beads were spun down at 500 x g for 5 min and washed with dilution buffer twice. Samples were eluted by 1 x Laemmli buffer and analyzed by Western blotting.

### Proteomics analysis

Samples for proteomic analysis were obtained from Hela cells transfected with control or the FAT1 intracellular part (amino acids 4203–4588) fused with the extracellular part and transmembrane domain of the IL-2 receptor (amino acids 1–259) and carrying a Flag tag on the C terminus for 36 h followed by lysis in IP buffer. Lysates were incubated with magnetic anti-FLAG beads (catalog #A8592, Sigma) overnight at 4 °C. Affinity purified samples were subjected to off-bead digest as described [43]. Resulting peptides were subjected to electrospray ionization (ESI)-mediated liquid chromatography/tandem mass spectrometry (LC/MS2) using in house-packed column emitters (15 cm length, 70 µm ID, 1.9 µm ReprsoSil-Pur 120 C18-AQ, Dr. Maisch) and a buffer system comprising solvent A (0.1% formic acid) and solvent B (80% acetonitrile, 0.1% formic acid). Instrumentation details and parameters were extracted and summarized using MARMoSET [44]. Peptide/spectrum matching and label free quantitation were performed using the MaxQuant suite of algorithms [45–47] against the human UniProt database (downloaded on 2021/02/08; 194237 entries). Downstream data analysis was performed with the in-house developed limma-based R autonomics [48].

### EdU incorporation analysis

Cells were seeded and transfected with siRNAs against the indicated genes. 24 hours after transfection, EdU was added to the cell culture medium at a final concentration of 10 µM. After an incubation period of 2–6 hours, cells were stained with DAPI and EdU using Click-iT^TM^ EdU Alexa Fluor^TM^ 647 flow cytometry assay kit (catalog #C10424) and were then analyzed by flow cytometry using a FACS Canto II (Becton Dickinson). Flow cytometric data were analyzed by FlowJo software (Tree Star Inc).

### Generation of stable FAT1^ICD^ overexpression and stable shRNA knockdown cells

Lentiviral expression vectors encoding the intracellular domain of FAT1 (amino acid 4203–4588) fused with extracellular and transmembrane domains (amino acids 1–259) of the human interleukin-2 receptor and vectors encoding scrambled shRNA or shRNA directed against human *FAT1* or *MIB2* were obtained from VectorBuilder (pLV[shRNA]-mCherry:T2A:Bsd-U6 > shScramble, pLV[shRNA]-mCherry:T2A:Bsd-U6 > shFAT1, pLV[shRNA]-mCherry:T2A:Bsd-U6 > shMIB2). Different vectors were transfected individually into Platinum-E cells along with the envelope plasmid pMD2.G and the packaging plasmid psPAX2 using Lipofectamine 3000^TM^ transfection reagent (Thermo Fisher Scientific) according to the manufacturer’s protocol. After 48 hours of incubation, lentiviral particle-rich supernatants were harvested, filtered through a 0.45 μm low-protein-binding Durapore membrane (Millex) and concentrated using Lenti-X-Concentrator (TakaraBio). For lentiviral transduction, target cells were seeded in 6-well plates, and the concentrated lentivirus was added. 48 hours post infection, cells were subjected to selection (8 µg/ml blasticidin or 10 µg/ml puromycin (Sigma)) for several days and were used for further analyses after being tested to be virus-free. Knock-down efficiency was determined by Western blotting. The target sequences of shRNAs were as follows: Scramble: 5-CCTAAGGTTAAGTCGCCCTCG-3; human FAT1: 5-AGATGCCGACGCAGGATTAAA-3; human MIB2_1: 5-AGGTGGTCGTCAGCAAGAAAC-3 (CAL27 cells); human MIB2_2: 5- GCGCTAGCTGTGAGAAAGATT-3 (Hela and CAL33 cells).

### Analysis of *in vitro* tumor cell growth

Cells were seeded on 48 or 96 well plates, and siRNA-mediated knock-down was performed on the following day. Medium was changed to DMEM medium 6 hours after transfection and time was set as 0h. Cell growth was monitored for the indicated time periods using the IncuCyte®ZOOM system (Essen Bioscience, Ann Arbor, MI, USA), which allows for the automated real time imaging of live cells. Growth rates were calculated as a percentage of cell confluence per image and over time.

### *In vivo* tumor growth

For *in vivo* tumor growth, 10^6^ tumor cells in 100 µl PBS were injected subcutaneously into the flank of 6–8 week old male (Hela cells) or female NOG mice (CAL27) or female nude mice (CAL33). Tumor growth was then monitored. Tumor growth was monitored three times per week or every two days using calipers, and all tumor volumes were kept within 1.7 cm³ as approved by the local animal ethics committee. Mice were euthanized no later than 30 days after tumor injection to examine primary tumors. Depending on tissue processing requirements, animals were sacrificed by cervical dislocation or CO₂ asphyxiation followed by thorax opening or by transcardial perfusion under deep anesthesia. To ensure animal welfare, a detailed scoring system was applied to monitor health and minimize suffering. Animal activity and mobility were assessed, with normal exploratory and free movement scoring 0, reduced spontaneous movement scoring 1, and conditions such as haggard flanks, minimal response to stimuli, or abnormal posture resulting in the immediate euthanasia. Fur condition and grooming activity were also evaluated, with normal, clean, shiny fur scoring 0, and reduced grooming or piloerection scoring 1. Mice were assessed at least twice weekly, and those with a cumulative score exceeding 5 or meeting other humane endpoint criteria were euthanized immediately. Humane endpoints included tumor size exceeding 1.7 cm^3^, evidence of ulceration, necrosis, or infection, significant weight loss (>20% of baseline), or signs of systemic illness or distress such as reduced grooming, lethargy, or isolation. All procedures were conducted under anesthesia (isoflurane, 2–3% for induction and 1–2% for maintenance) to ensure animal comfort, and analgesics were administered when necessary to alleviate discomfort unrelated to tumor progression. Animals were housed in individually ventilated cages under a 12-hour light/dark cycle with ad libitum access to food and water. The number of animals used for each experiment is given in the Results section and the corresponding figures. In our experiments, no animals were lost to premature death as a result of the experiment.

Research staff involved in animal care and handling received specialized training in accordance with institutional and national guidelines to ensure proper monitoring and handling of animals throughout the experiment. This training included techniques for administering anesthesia, scoring animal welfare, and performing humane euthanasia procedures. NOG mice (NOD.Cg-Prkdc^scid^ Il2rg^tm^ 1^Sug^/JicTac) and nude mice (Rj:ATHYM-Foxn1nu/nu) were obtained from Taconic and janvier labs respectively.

All procedures involving animal care and use in this study were approved by the local animal ethics committee (Regierungspräsidium Darmstadt, Germany) with the approval number: B2/2051 and B2/1175.

All experiments were performed in accordance with the relevant guidelines and regulations.

### Statistics

Statistical analyses were carried out using GraphPad Prism version 9.3.1 (GraphPad Software Inc., La Jolla, CA, USA). Results are shown as mean ± SEM, with the number of independent experiments represented by “n”. For comparisons involving two groups, an unpaired two-tailed Student’s t-test was applied. For data involving multiple groups, one-way ANOVA followed by Tukey’s post-hoc test was utilized unless specified otherwise. Time-dependent comparisons across multiple groups were analyzed using two-way ANOVA with Bonferroni’s correction. A *p*-value below 0.05 was regarded as statistically significant.

## Supporting information

S1 Fig Loss of FAT1 expression results in increased YAP/TAZ protein levels in MDA-MB-231 and U-87 cells. (A-D) MDA-MB-231 (A, C) and U-87 (B, D) cells were transfected with control siRNA or siRNA directed against *FAT1*. Thereafter, the protein levels of YAP and TAZ were analyzed by immunoblotting (A, B) or the expression of the indicated YAP/TAZ target genes as well as of *FAT1* was determined by RT-qPCR (C, D). Shown is a representative of 3 independently performed experiments (A, B) (n = 3 in A-D). Data are presented as mean values ± SEM. *, P ≤ 0.05; **, P ≤ 0.01; ***, P ≤ 0.001 (two-tailed unpaired t-test).
(TIF)

S2 Fig Analysis of HNSCCs. (A) The indicated HNSCCs were analyzed for the expression of FAT1, MIB2, CAMK2 and YAP/TAZ using immunoblotting. Shown is a representative of 3 independently performed experiments.
(TIF)

S3 Fig FAT1 controls degradation of YAP and TAZ proteins in MDA-MB-231 and U-87 cells. (A, B) YAP and TAZ protein degradation in MDA-MB-231 (A) and in U-87 (B) cells transfected with control siRNA or siRNA directed against *FAT1* was analyzed after incubation of cells with 50 µg/ml cycloheximide (CHX) for the indicated time periods. Shown are the YAP and TAZ protein levels as determined by immunoblotting. Diagrams show the statistical evaluation (n = 3 independently performed experiments). Data are normalized to the basal levels of YAP and TAZ at time point 0. Data are represented as mean values ± SEM. *, P ≤ 0.05; ***, P ≤ 0.001 (two-way ANOVA plus Bonferroni’s post-hoc test).
(TIF)

S4 Fig Effect of MIB2 knock-down on YAP/TAZ protein levels and gene expression in MDA-MB-231 and U-87 cells. (A-D) MDA-MB-231 cells (A, B) and U-87 cells (C, D) were transfected with control siRNA or siRNA directed against *MIB2*. Thereafter, the protein levels of YAP and TAZ were analyzed by immunoblotting (A, C) or the expression of the indicated YAP/TAZ target genes as well as of *MIB2* were determined by RT-qPCR (B, D). Shown is a representative of 3 independently performed experiments with the statistical analysis (A, C) (n = 3 in A-D). Data are represented as mean values ± SEM. *, P ≤ 0.05; **, P ≤ 0.01; ***, P ≤ 0.001 (two-tailed unpaired t-test).
(TIF)

S5 Fig FAT1 controls degradation of YAP and TAZ proteins in HN13 and CAL27 cells. HN13 (A) and CAL27 cells (B) were transfected with control siRNA or siRNA directed against *MIB2*. Cells were then treated in the absence and presence of 50 µg/ml CHX for the indicated time periods, and the protein levels of YAP and TAZ were analyzed by immunoblotting. Shown is a representative of 3 independently performed experiments with the statistical analysis (n = 3). Data are normalized to the basal levels of YAP and TAZ at time point 0. Data are represented as mean values ± SEM. *, P ≤ 0.05; **, P ≤ 0.01; ***, P ≤ 0.001 (two-way ANOVA plus Bonferroni’s post-hoc test).
(TIF)

S6 Fig Role of YAP/TAZ in FAT1-regulated HNSCC growth. (A,B) CAL27 cells, which express endogenously FAT1, were transfected with control siRNA or siRNA directed against the indicated RNAs, and EdU incorporation (A) or cell growth (B) were analyzed (n = 3). (C, D) CAL33 cells, which lack FAT1, were transfected with control plasmid or with eukaryotic expression plasmids bearing the cDNA of the FAT1 intracellular domain (FAT1^ICD^) or YAP alone (YAP^OE^) or together. Thereafter, EdU incorporation (C) or cell proliferation (D) was analyzed (n = 3). Shown are mean values ± S.E.M; *, P ≤ 0.05; **, P ≤ 0.01; ***, P ≤ 0.001; n.s., non-significant (one-way ANOVA and Tukey’s post-hoc test (A, C) and two-way ANOVA and Bonferroni’s post-hoc test (B, D). Statistical comparisons in S6B Fig are labelled as follows: (a) siCtrl vs siFAT1; (b) siCtrl vs siMIB2; (c) siCtrl vs siYAP/TAZ, siCtrl vs siFAT1/YAP/TAZ and siCtrl vs siMIB2/YAP/TAZ. Statistical comparisons in S6D Fig are labelled as follows: (a) Control vs YAP^OE^ and Control vs FAT1^ICD^+YAP^OE^; (b) Control vs FAT1^ICD^.
(TIF)

S7 Fig Efficiency of shRNA-mediated knock-down of FAT1 or MIB2 in different tumor cells. (A-C) Hela cells (A) and CAL27 cells (B) transduced with control shRNA or shRNA directed against *FAT1* or *MIB2* and CAL33 cells (C) expressing FAT1^ICD^ or not (control) and transduced with control shRNA or shRNA directed against *MIB2* were analyzed by immunoblotting for expression of FAT1 or MIB2. Analysis of expression of GAPDH served as a control.
(TIF)

S1 Table Proteins enriched by immunoprecipitation of FAT^ICD^ from HeLa cell lysates. The proteomics data were statistically analyzed using a two-sided Bayesian moderated t-test implemented in the *limma* package.
(TIF)

S1 Raw Images Uncropped original Western blot images corresponding to the figures presented in the manuscript. (PDF)

S1 Data Source data file for Manuscript #PONE-D-24-52574. (XLSX)
