# Supplementary figures and images for: The tumor suppressor FAT1 controls YAP/TAZ protein degradation and tumor cell proliferation through E3 ligase MIB2
Source: PLoS One. 2025 Jun 6;20(6):e0325535. doi: 10.1371/journal.pone.0325535 (PMC12143506; doi:10.1371/journal.pone.0325535)

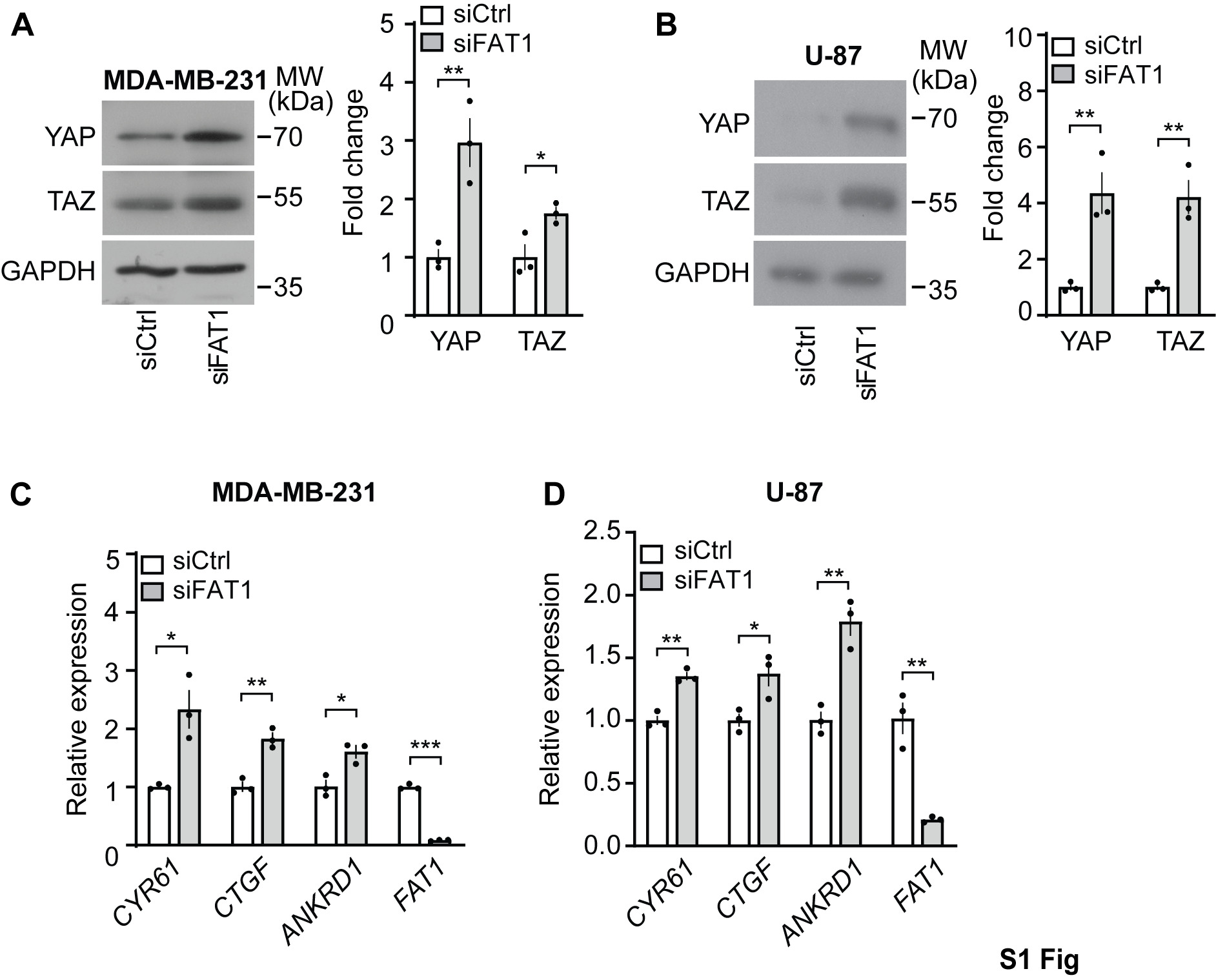

Supplement: S1 Fig — (A-D) MDA-MB-231 (A, C) and U-87 (B, D) cells were transfected with control siRNA or siRNA directed against FAT1. Thereafter, the protein levels of YAP and TAZ were analyzed by immunoblotting (A, B) or the expression of the indicated YAP/TAZ target genes as well as of FAT1 was determined by RT-qPCR (C, D). Shown is a representative of 3 independently performed experiments (A, B) (n = 3 in A-D). Data are presented as mean values ± SEM. *, P ≤ 0.05; **, P ≤ 0.01; ***, P ≤ 0.001 (two-tailed unpaired t-test). (TIF) [file pone.0325535.s001.tif]

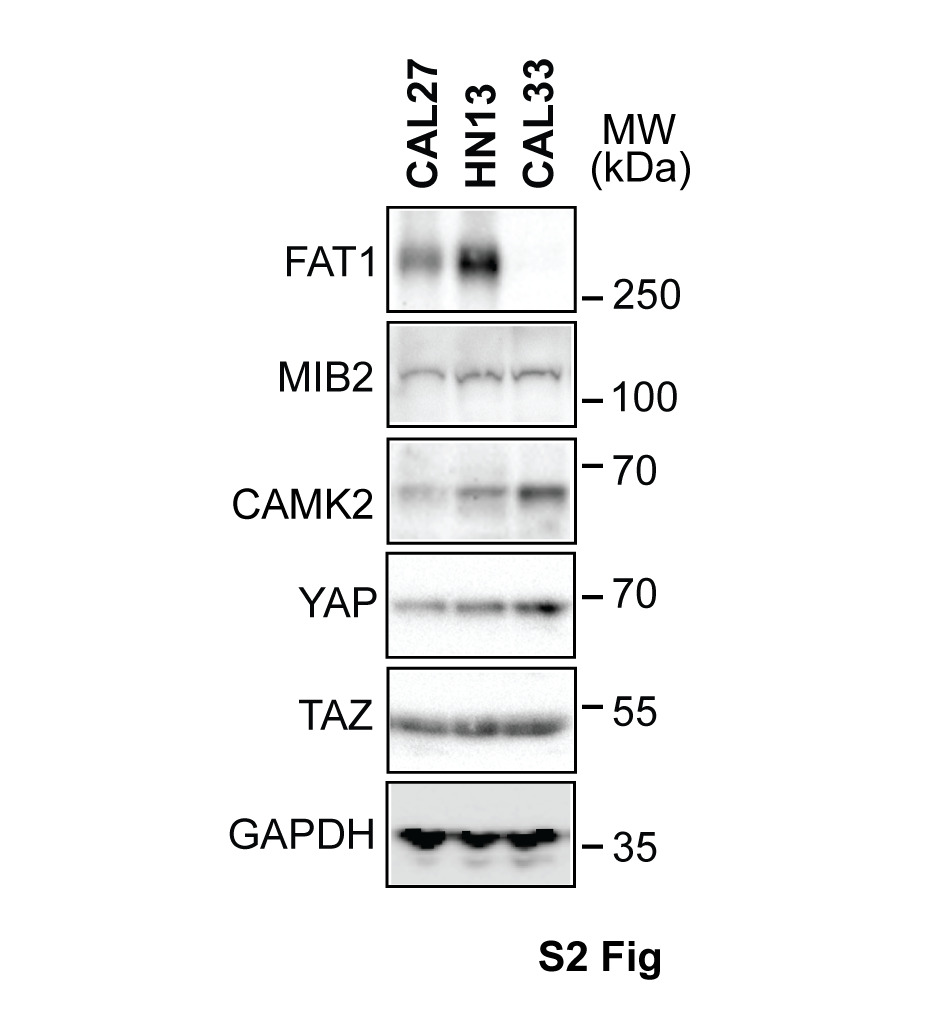

Supplement: S2 Fig — (A) The indicated HNSCCs were analyzed for the expression of FAT1, MIB2, CAMK2 and YAP/TAZ using immunoblotting. Shown is a representative of 3 independently performed experiments. (TIF) [file pone.0325535.s002.tif]

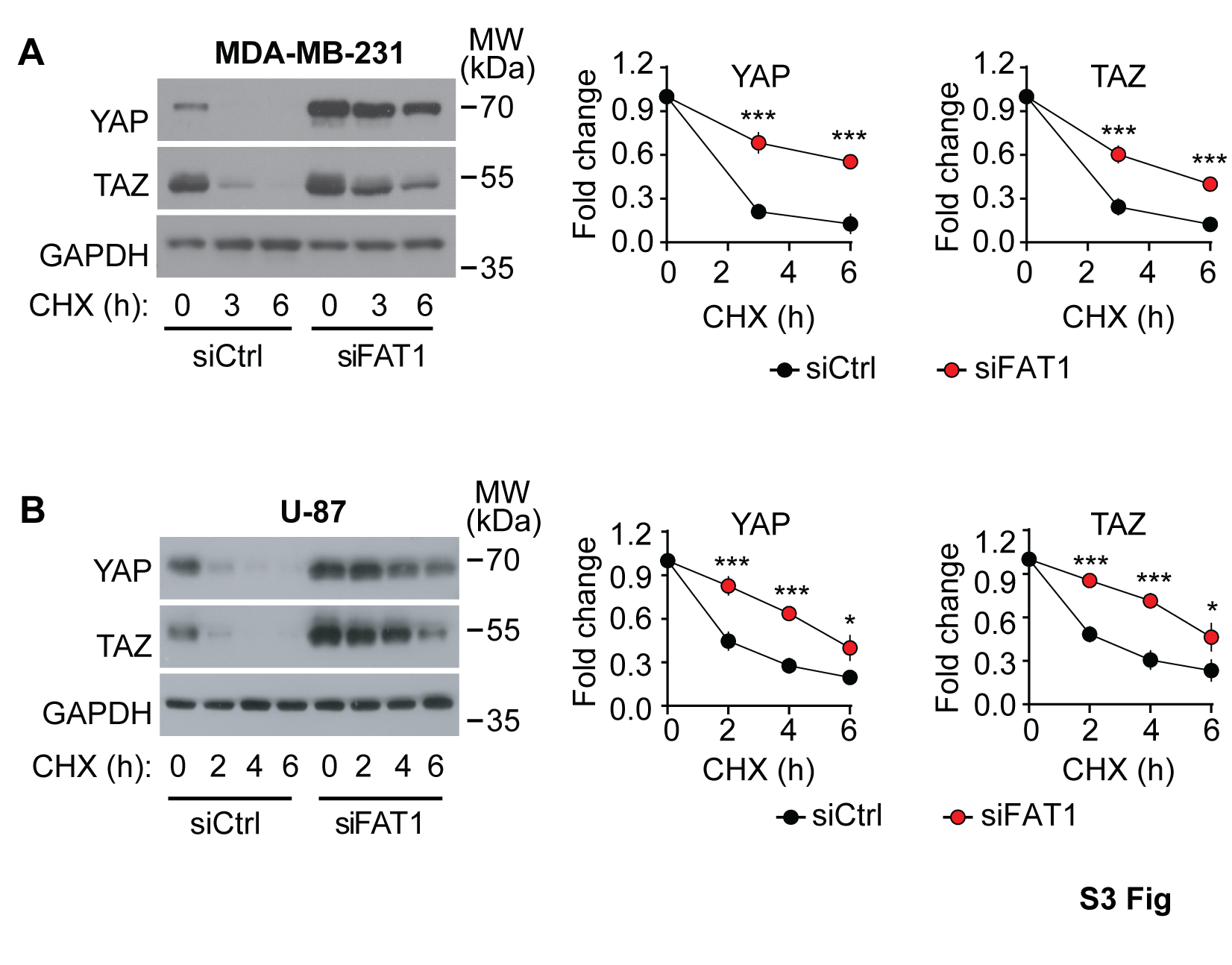

Supplement: S3 Fig — (A, B) YAP and TAZ protein degradation in MDA-MB-231 (A) and in U-87 (B) cells transfected with control siRNA or siRNA directed against FAT1 was analyzed after incubation of cells with 50 µg/ml cycloheximide (CHX) for the indicated time periods. Shown are the YAP and TAZ protein levels as determined by immunoblotting. Diagrams show the statistical evaluation (n = 3 independently performed experiments). Data are normalized to the basal levels of YAP and TAZ at time point 0. Data are represented as mean values ± SEM. *, P ≤ 0.05; ***, P ≤ 0.001 (two-way ANOVA plus Bonferroni’s post-hoc test). (TIF) [file pone.0325535.s003.tif]

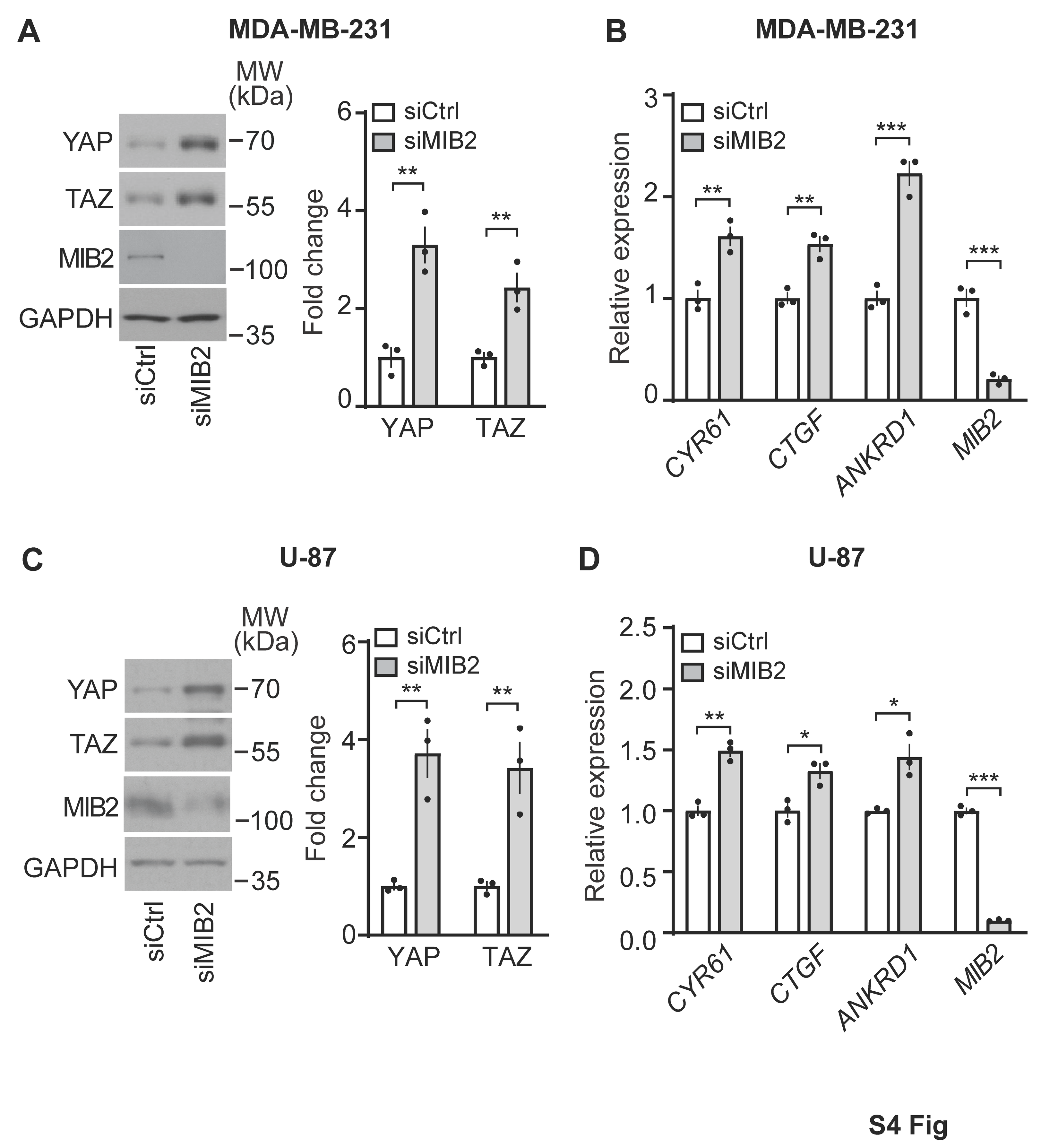

Supplement: S4 Fig — (A-D) MDA-MB-231 cells (A, B) and U-87 cells (C, D) were transfected with control siRNA or siRNA directed against MIB2. Thereafter, the protein levels of YAP and TAZ were analyzed by immunoblotting (A, C) or the expression of the indicated YAP/TAZ target genes as well as of MIB2 were determined by RT-qPCR (B, D). Shown is a representative of 3 independently performed experiments with the statistical analysis (A, C) (n = 3 in A-D). Data are represented as mean values ± SEM. *, P ≤ 0.05; **, P ≤ 0.01; ***, P ≤ 0.001 (two-tailed unpaired t-test). (TIF) [file pone.0325535.s004.tif]

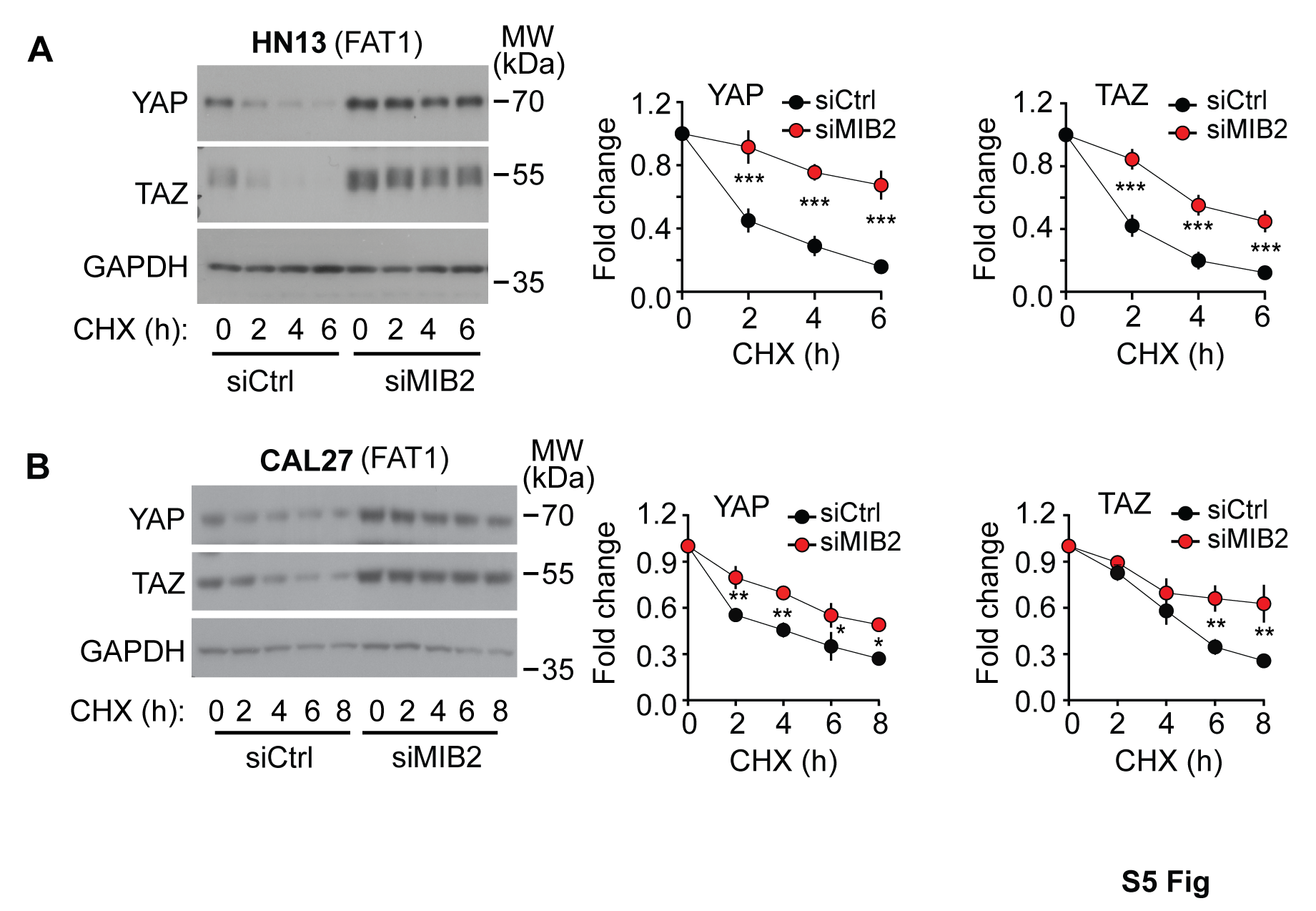

Supplement: S5 Fig — HN13 (A) and CAL27 cells (B) were transfected with control siRNA or siRNA directed against MIB2. Cells were then treated in the absence and presence of 50 µg/ml CHX for the indicated time periods, and the protein levels of YAP and TAZ were analyzed by immunoblotting. Shown is a representative of 3 independently performed experiments with the statistical analysis (n = 3). Data are normalized to the basal levels of YAP and TAZ at time point 0. Data are represented as mean values ± SEM. *, P ≤ 0.05; **, P ≤ 0.01; ***, P ≤ 0.001 (two-way ANOVA plus Bonferroni’s post-hoc test). (TIF) [file pone.0325535.s005.tif]

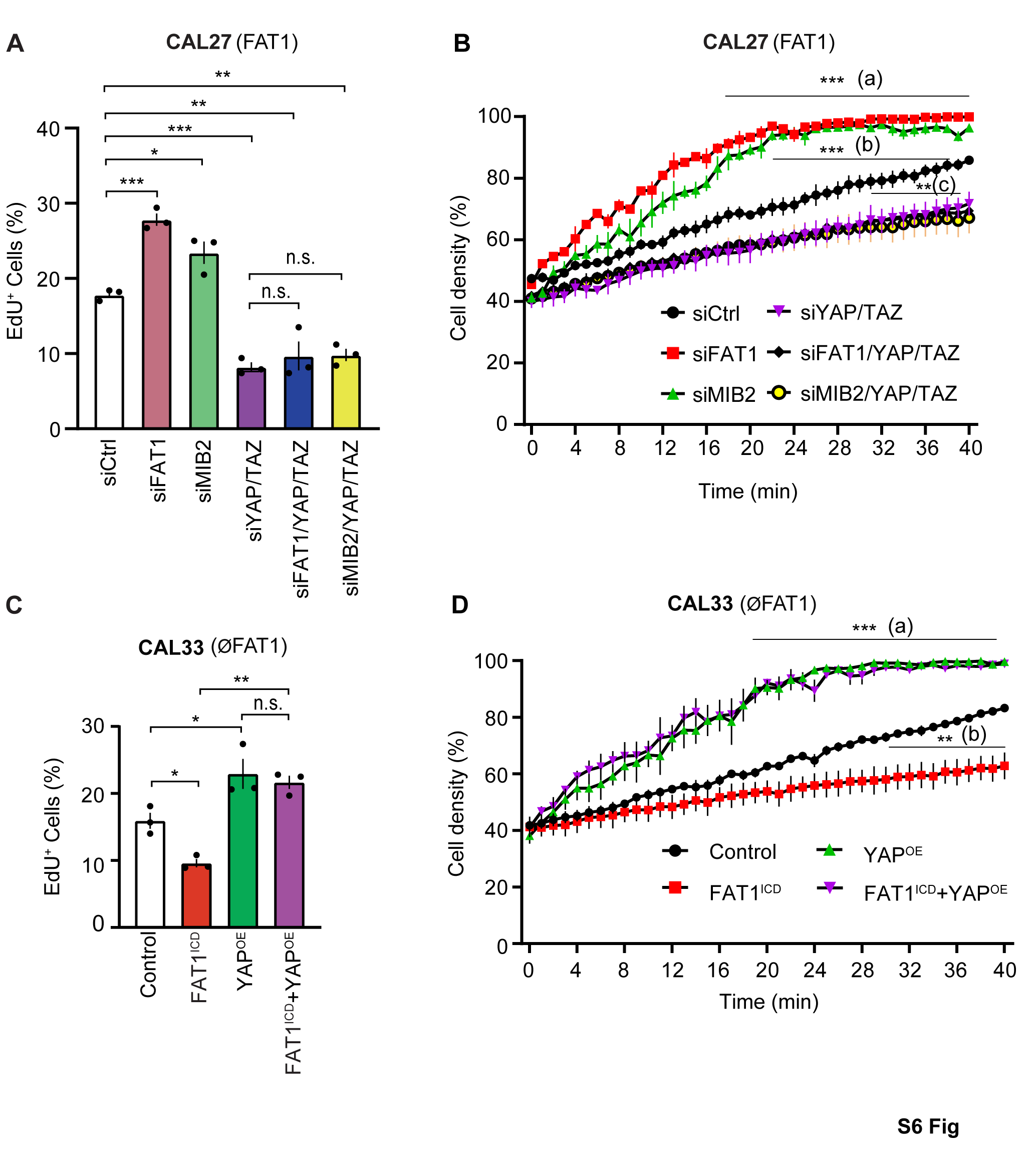

Supplement: S6 Fig — (A,B) CAL27 cells, which express endogenously FAT1, were transfected with control siRNA or siRNA directed against the indicated RNAs, and EdU incorporation (A) or cell growth (B) were analyzed (n = 3). (C, D) CAL33 cells, which lack FAT1, were transfected with control plasmid or with eukaryotic expression plasmids bearing the cDNA of the FAT1 intracellular domain (FAT1ICD) or YAP alone (YAPOE) or together. Thereafter, EdU incorporation (C) or cell proliferation (D) was analyzed (n = 3). Shown are mean values ± S.E.M; *, P ≤ 0.05; **, P ≤ 0.01; ***, P ≤ 0.001; n.s., non-significant (one-way ANOVA and Tukey’s post-hoc test (A, C) and two-way ANOVA and Bonferroni’s post-hoc test (B, D). Statistical comparisons in S6B Fig are labelled as follows: (a) siCtrl vs siFAT1; (b) siCtrl vs siMIB2; (c) siCtrl vs siYAP/TAZ, siCtrl vs siFAT1/YAP/TAZ and siCtrl vs siMIB2/YAP/TAZ. Statistical comparisons in S6D Fig are labelled as follows: (a) Control vs YAPOE and Control vs FAT1ICD+YAPOE; (b) Control vs FAT1ICD. (TIF) [file pone.0325535.s006.tif]

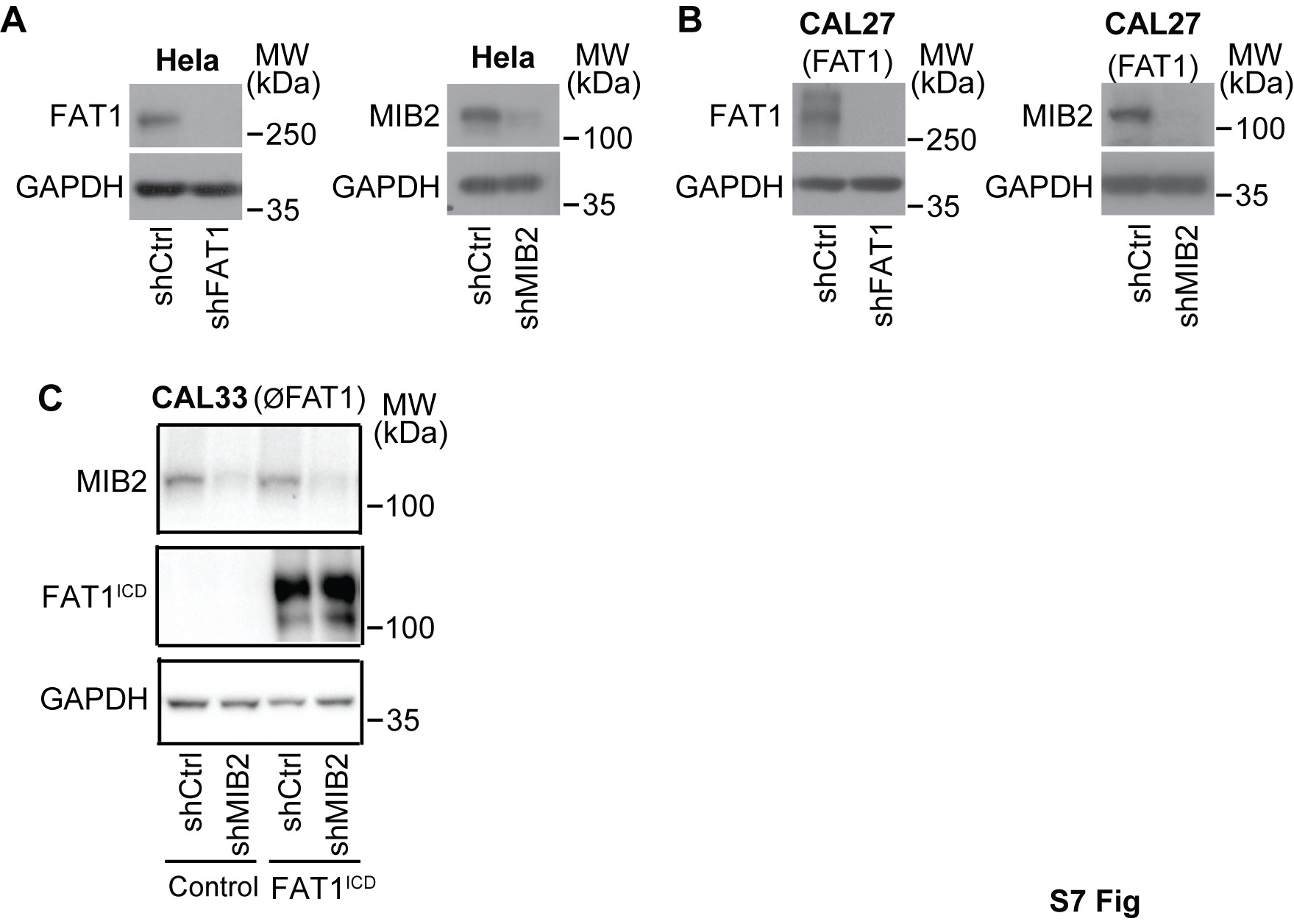

Supplement: S7 Fig — (A-C) Hela cells (A) and CAL27 cells (B) transduced with control shRNA or shRNA directed against FAT1 or MIB2 and CAL33 cells (C) expressing FAT1ICD or not (control) and transduced with control shRNA or shRNA directed against MIB2 were analyzed by immunoblotting for expression of FAT1 or MIB2. Analysis of expression of GAPDH served as a control. (TIF) [file pone.0325535.s007.tif]

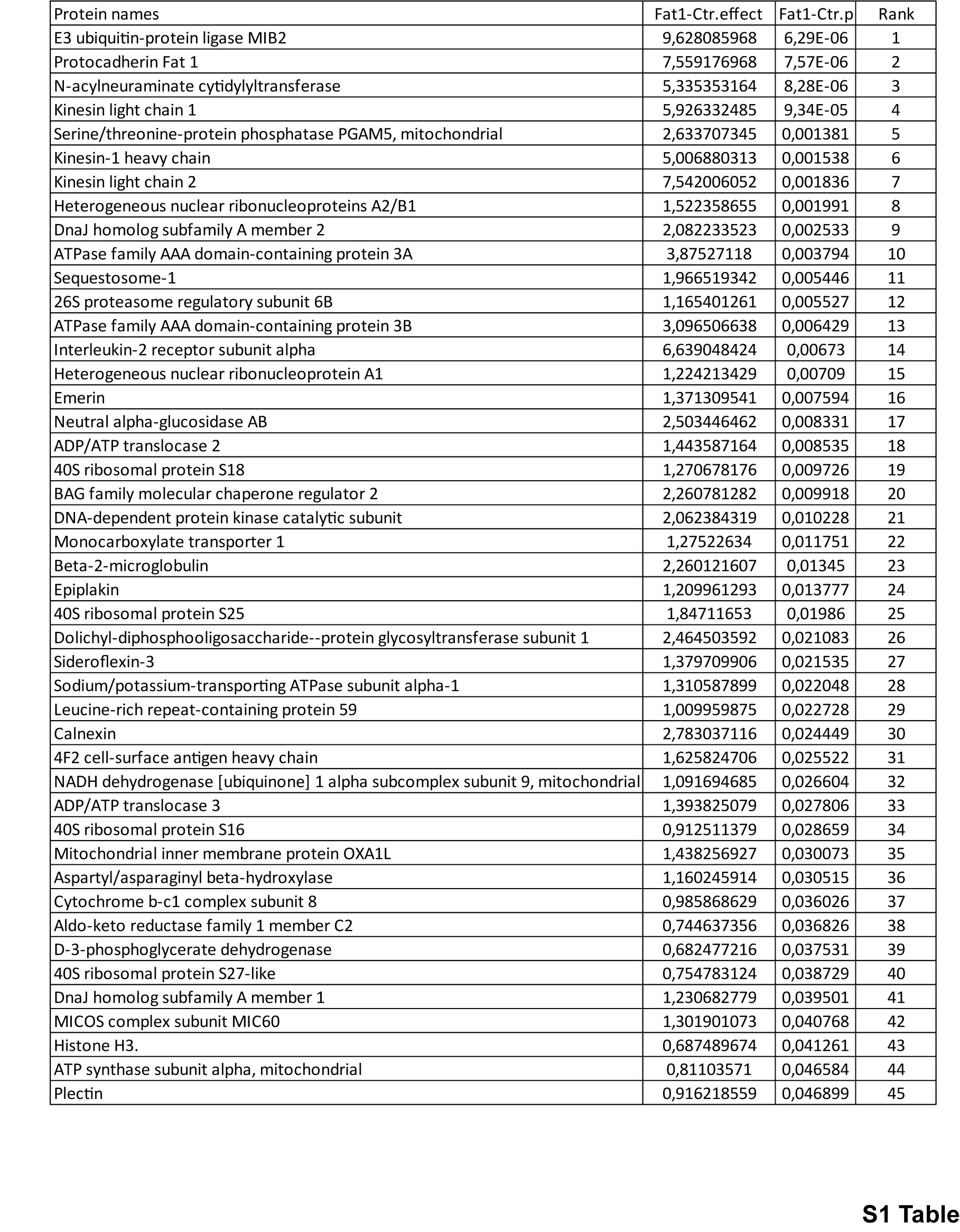

Supplement: S1 Table — The proteomics data were statistically analyzed using a two-sided Bayesian moderated t-test implemented in the limma package. (TIF) [file pone.0325535.s008.tif]
