# Supplementary material for: The tumor suppressor FAT1 controls YAP/TAZ protein degradation and tumor cell proliferation through E3 ligase MIB2
Source: PLoS One. 2025 Jun 6;20(6):e0325535. doi: 10.1371/journal.pone.0325535 (PMC12143506; doi:10.1371/journal.pone.0325535)
Supplement: S1 Raw Images — (PDF) [file pone.0325535.s009.pdf]

Raw images prepared in Figs. 1-5 and 7 as well as Suppl. Figs. 1-5 and 7

Fig.1A

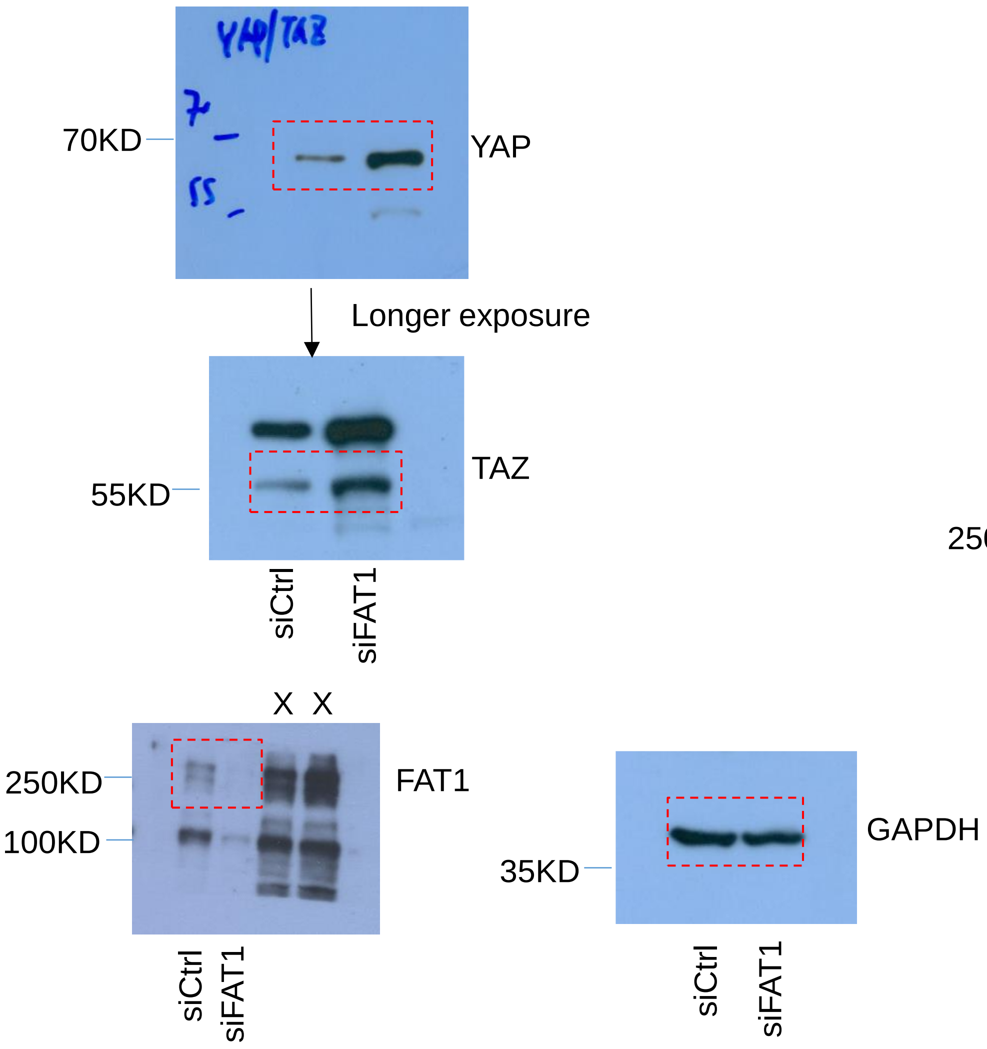

Fig.1D

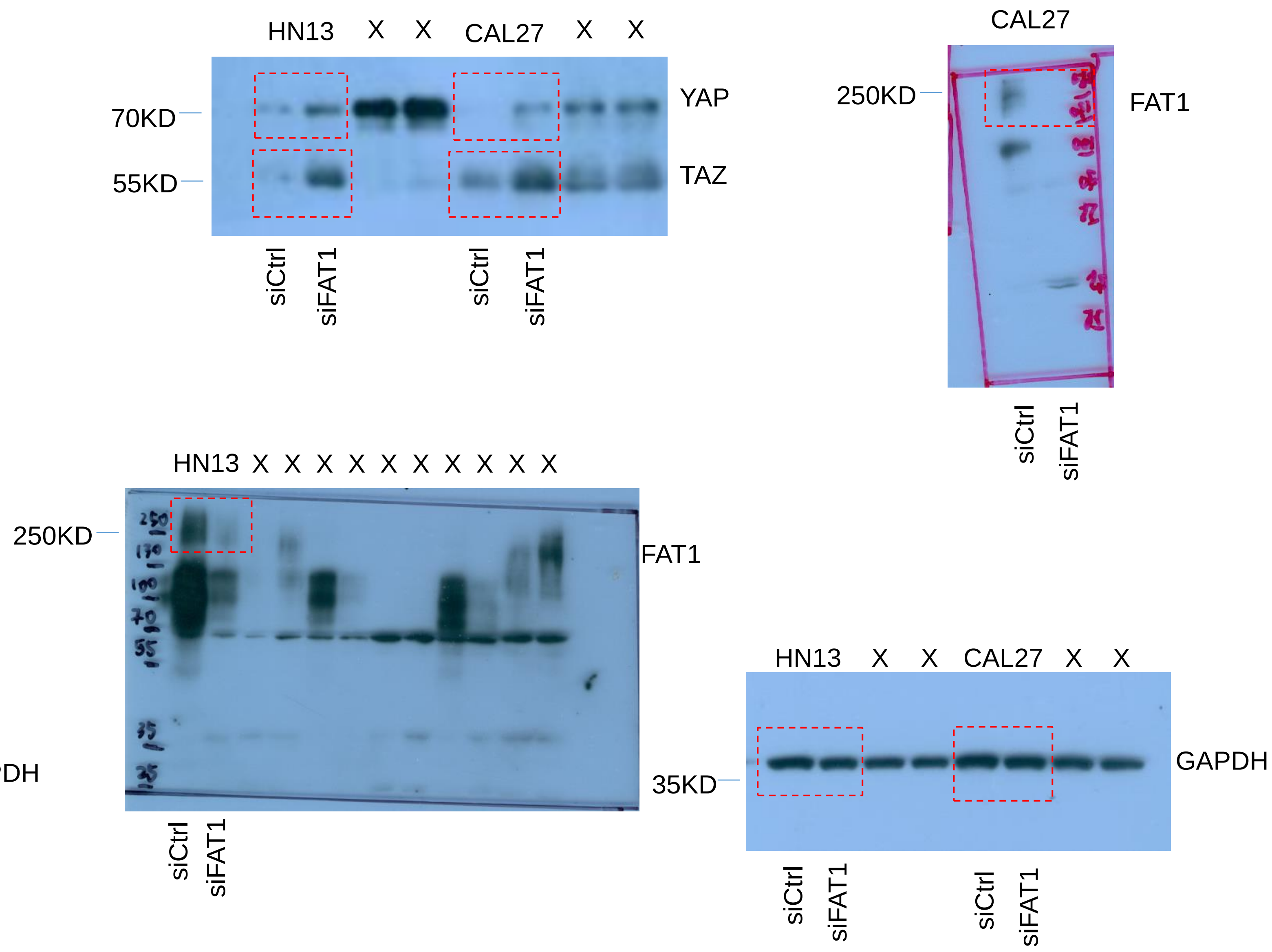

The blots shown here were obtained using film-based chemiluminescent detection.

Fig.1D

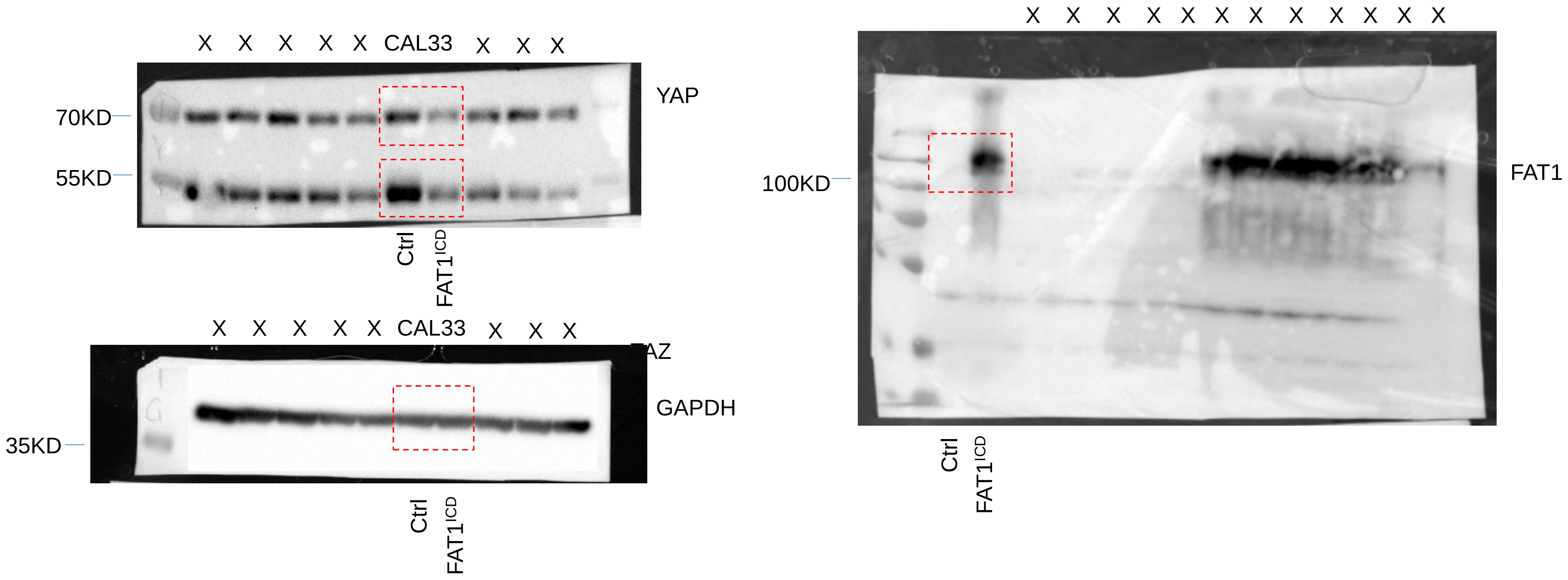

The blots shown here were obtained using ChemiDoc chemiluminescent Imaging.

Fig.2A

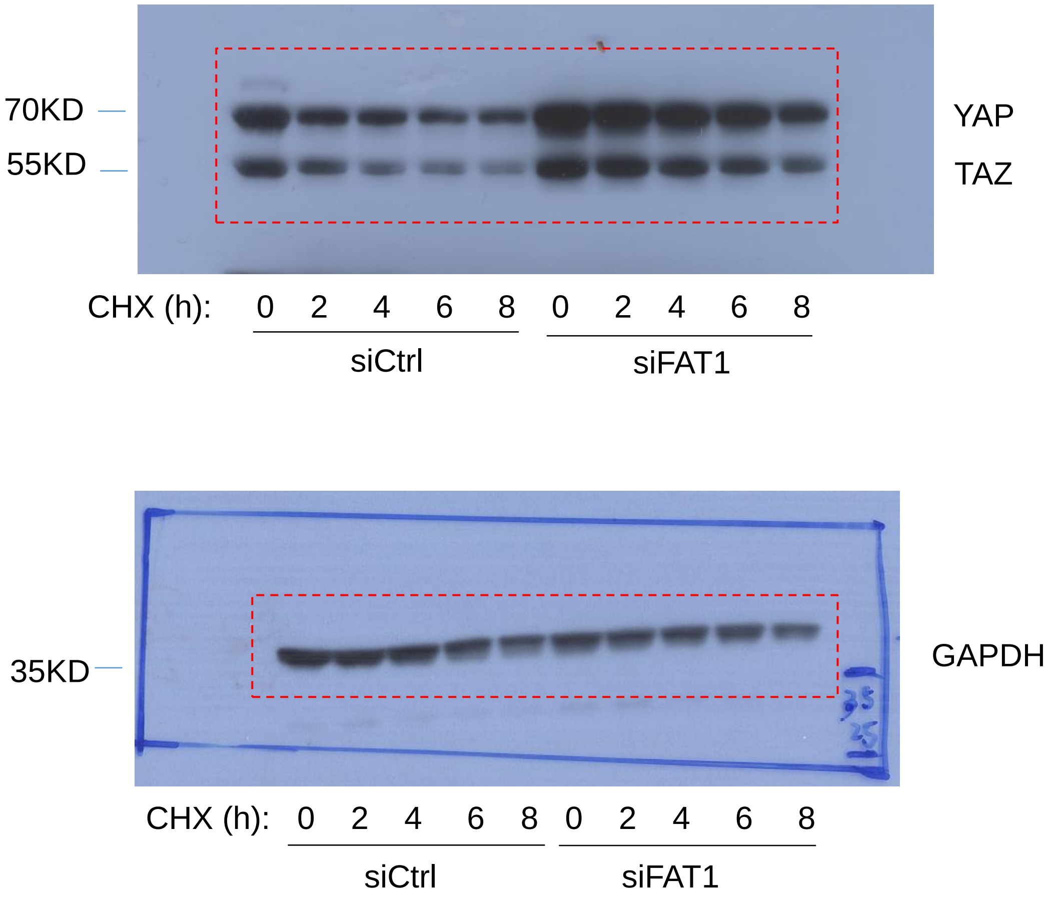

The blots shown left here were obtained using film-based chemiluminescent detection

Fig.2B

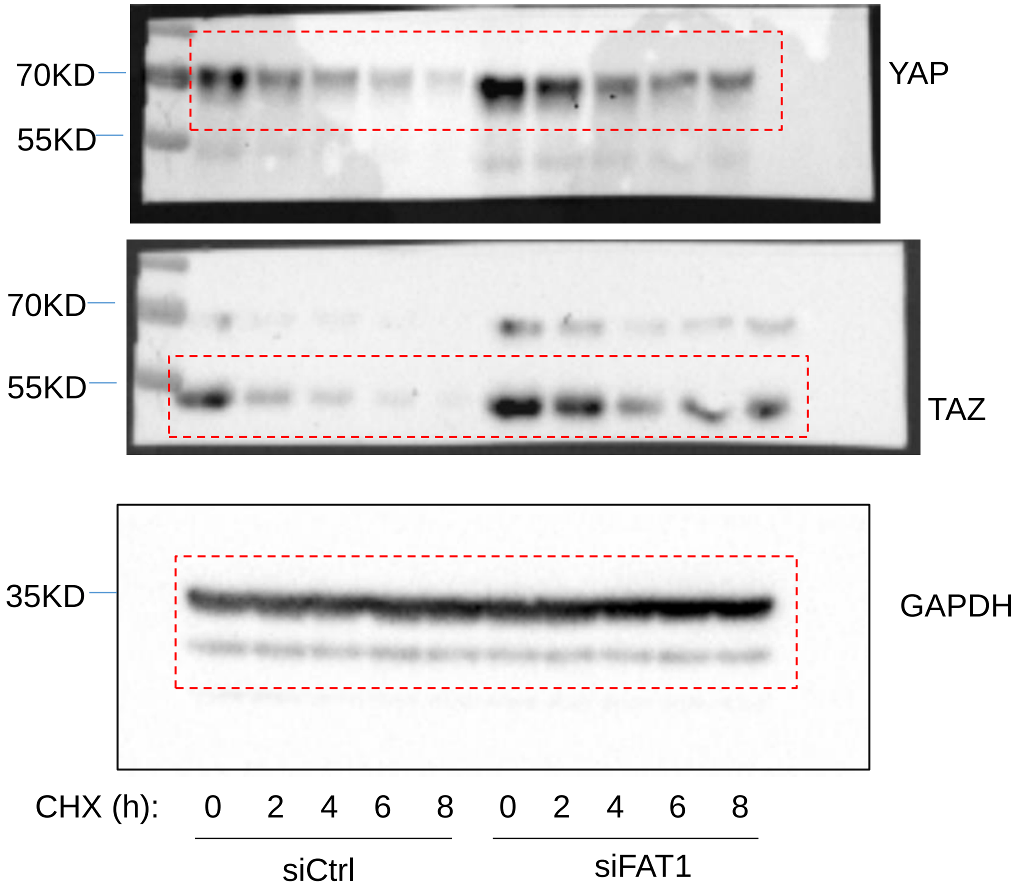

The blots shown right here were obtained using ChemiDoc chemiluminescent Imaging.

Fig. 2C

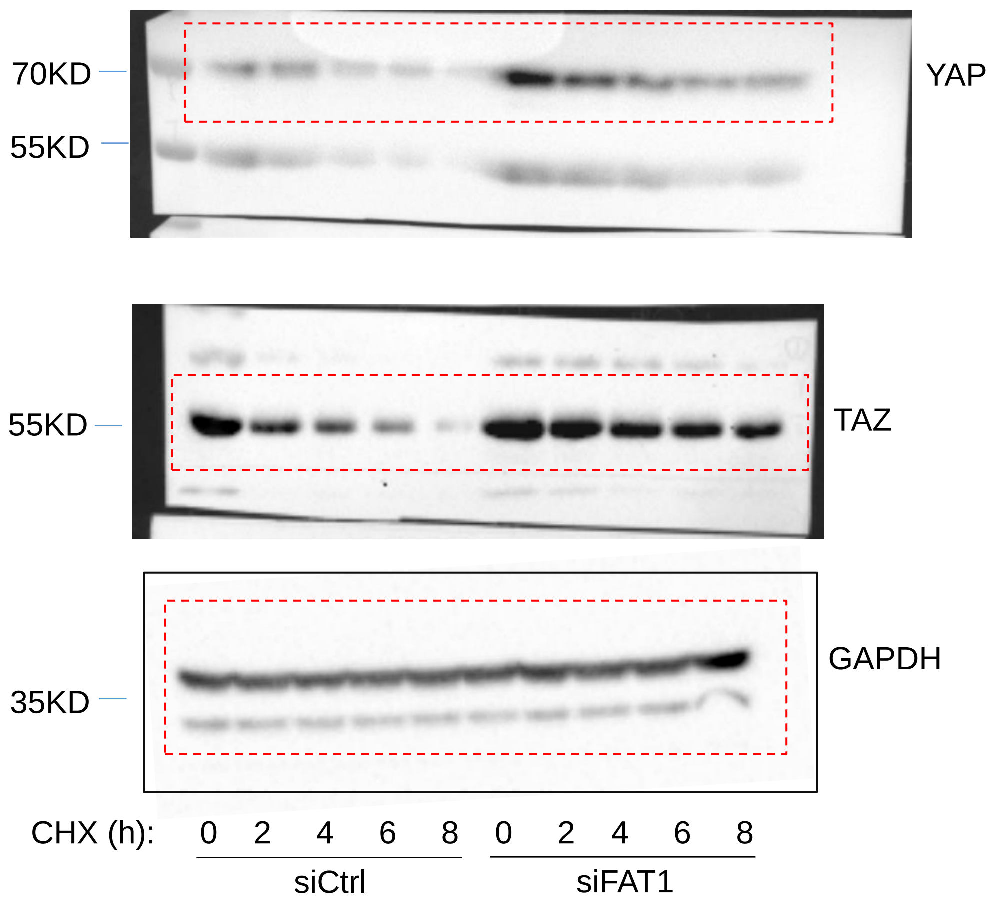

Fig. 2D

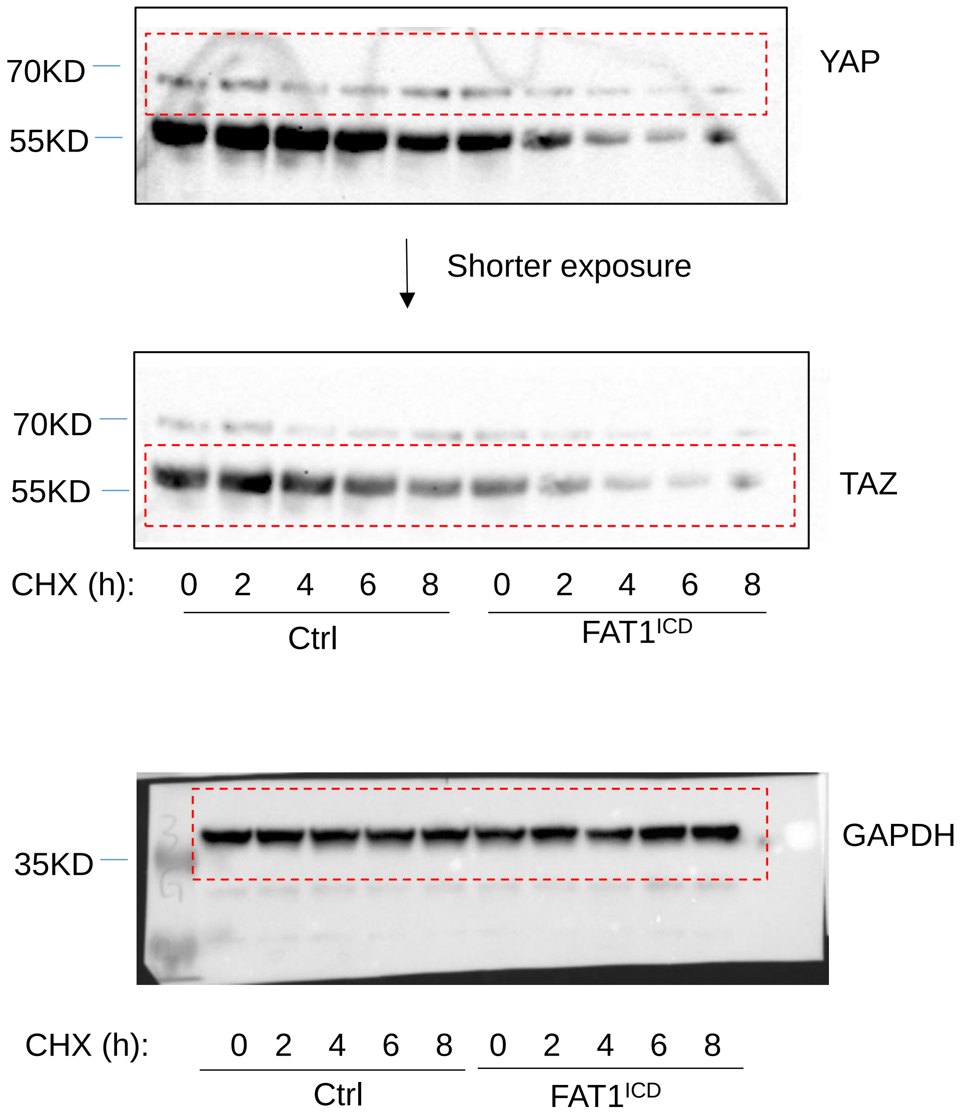

The blots shown here were obtained using ChemiDoc chemiluminescent Imaging.

Fig. 3A

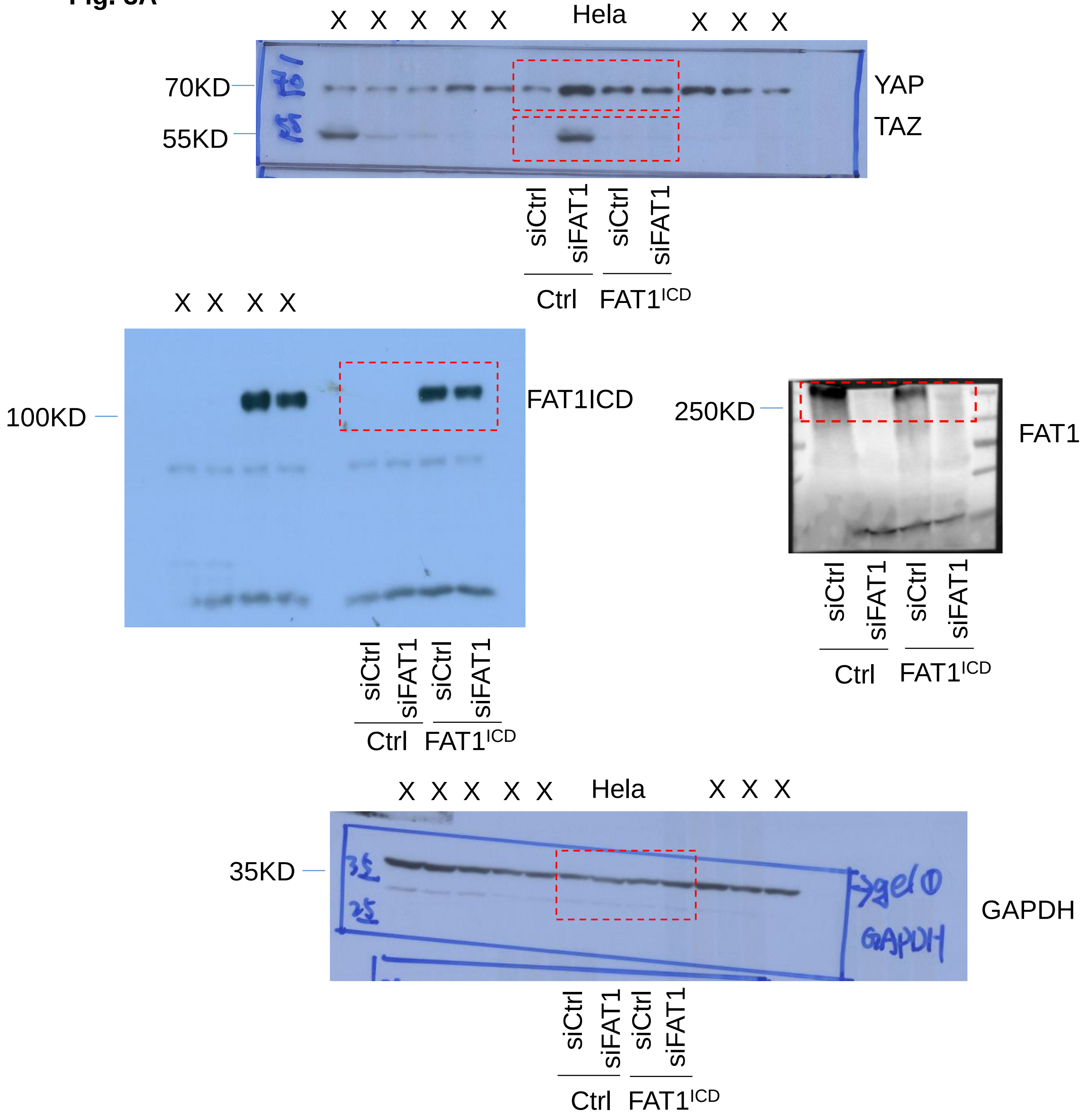

Fig. 3C

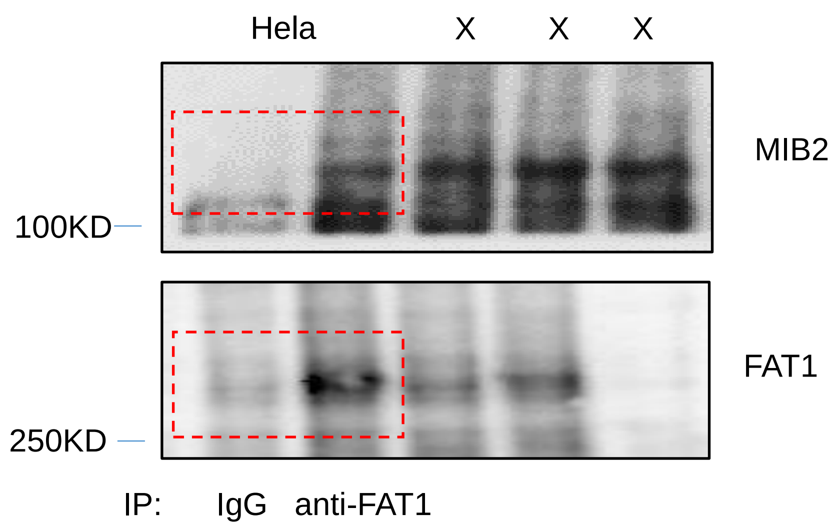

The blots shown in Fig. 3C and FAT1 blot in Fig. 3A were obtained using ChemiDoc chemiluminescent Imaging and the rest blots here were obtained using film-based chemiluminescent detection.

Fig. 3D

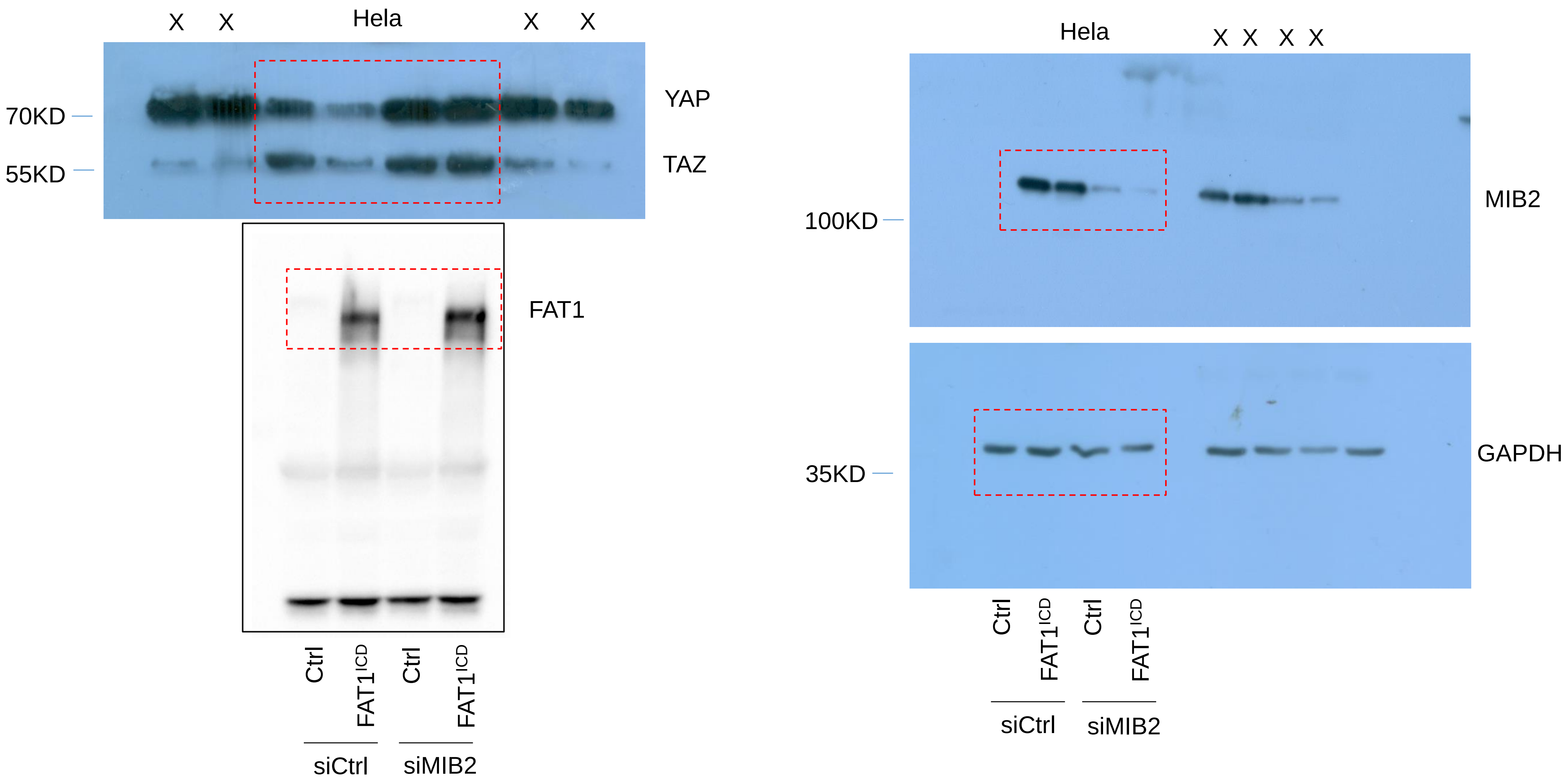

The FAT1 blot shown here was obtained using ChemiDoc chemiluminescent Imaging and the rest blots were obtained using film-based chemiluminescent detection.

Fig. 3F

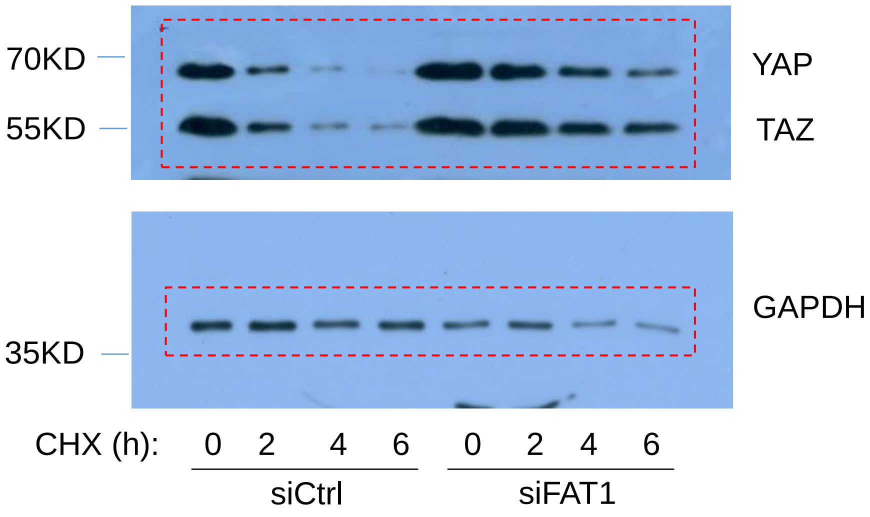

Fig. 3G

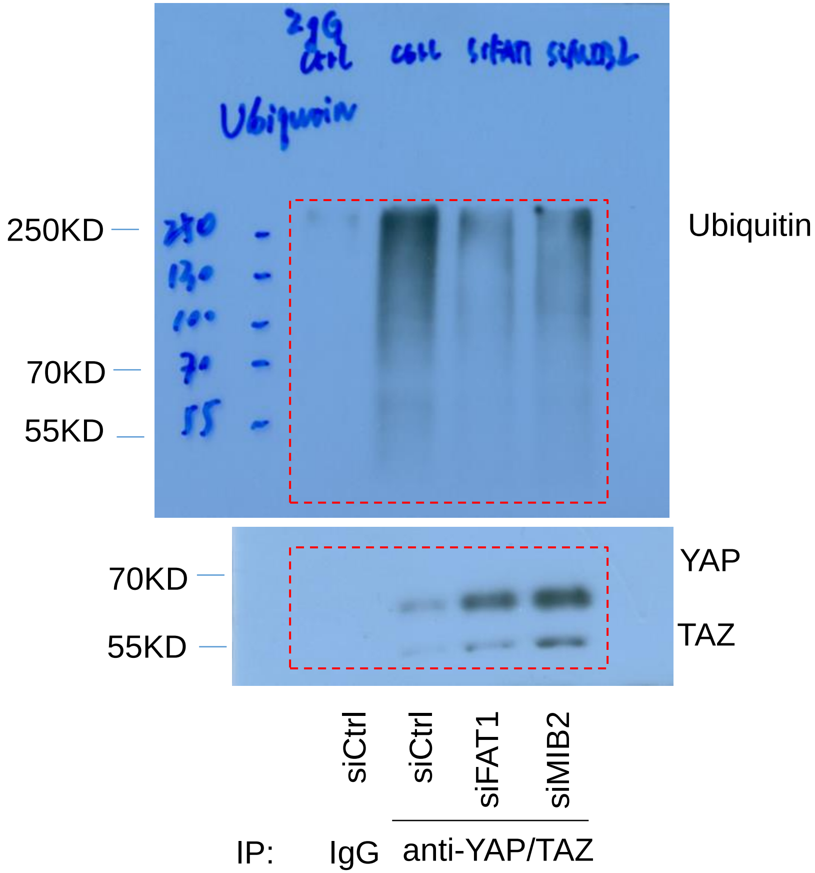

The blots shown here were obtained using film-based chemiluminescent detection.

Fig. 4A

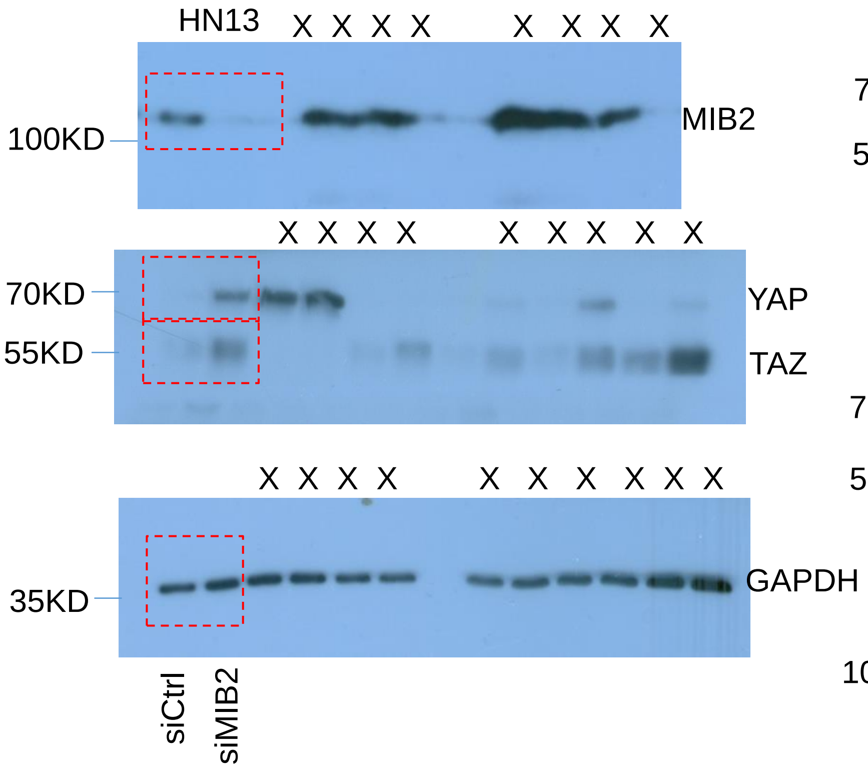

Fig. 4C

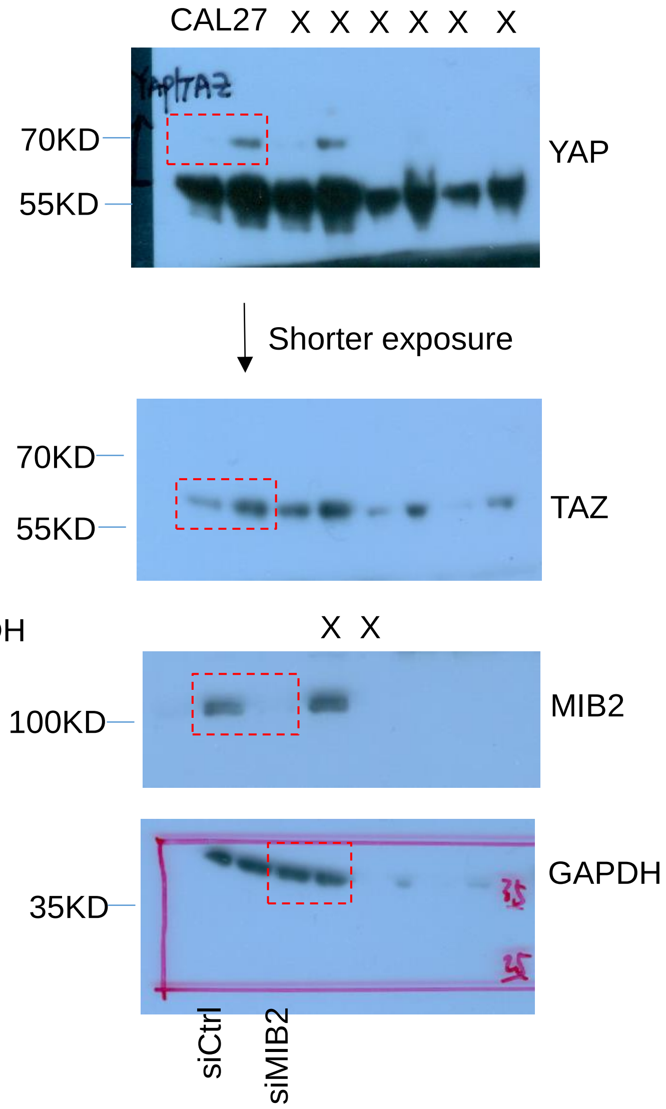

Fig. 4E

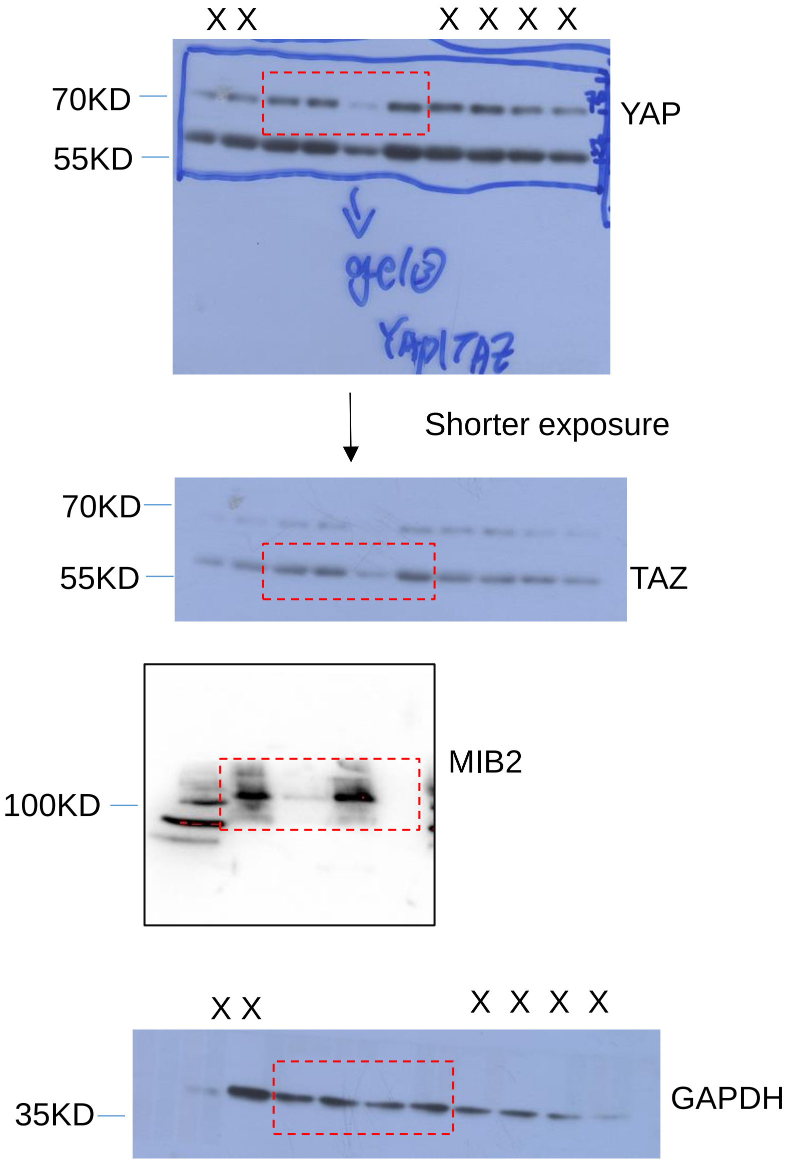

The blots shown here were obtained using film-based chemiluminescent detection.

Fig. 4 G

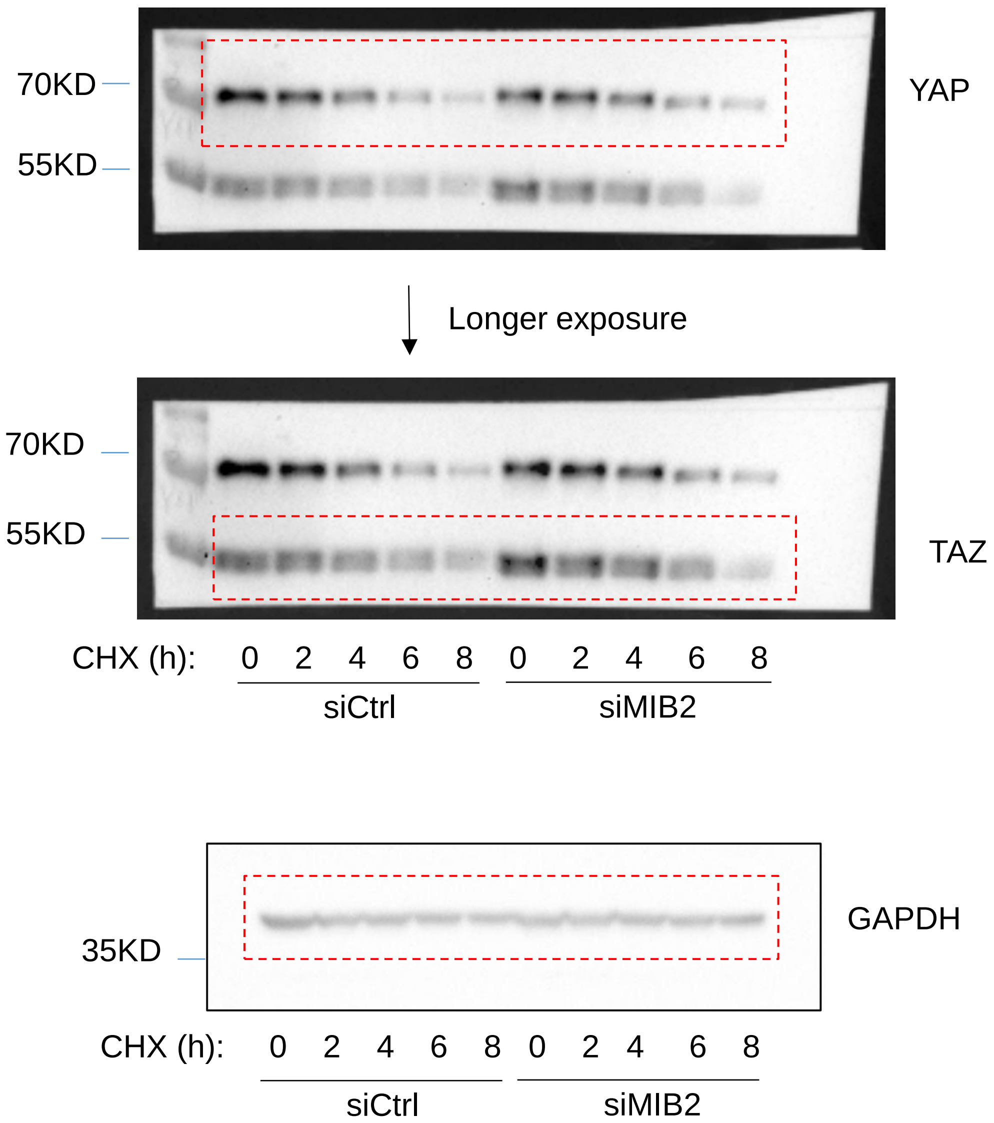

Fig. 4H

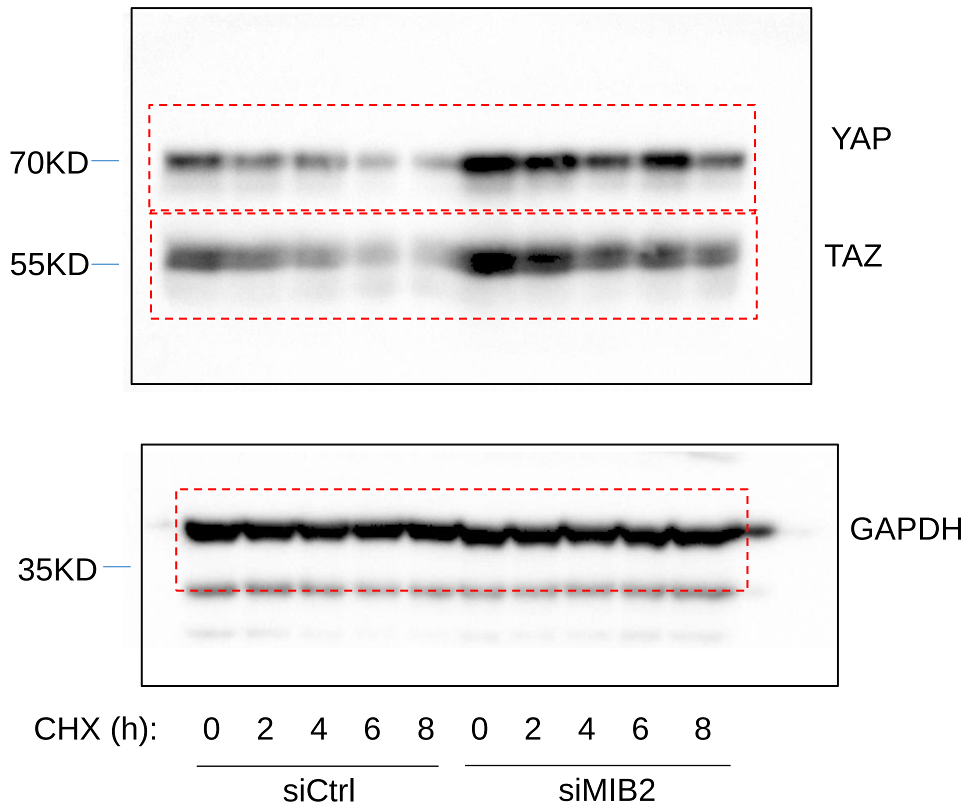

The blots shown here were obtained using ChemiDoc chemiluminescent Imaging.

Fig. 5A

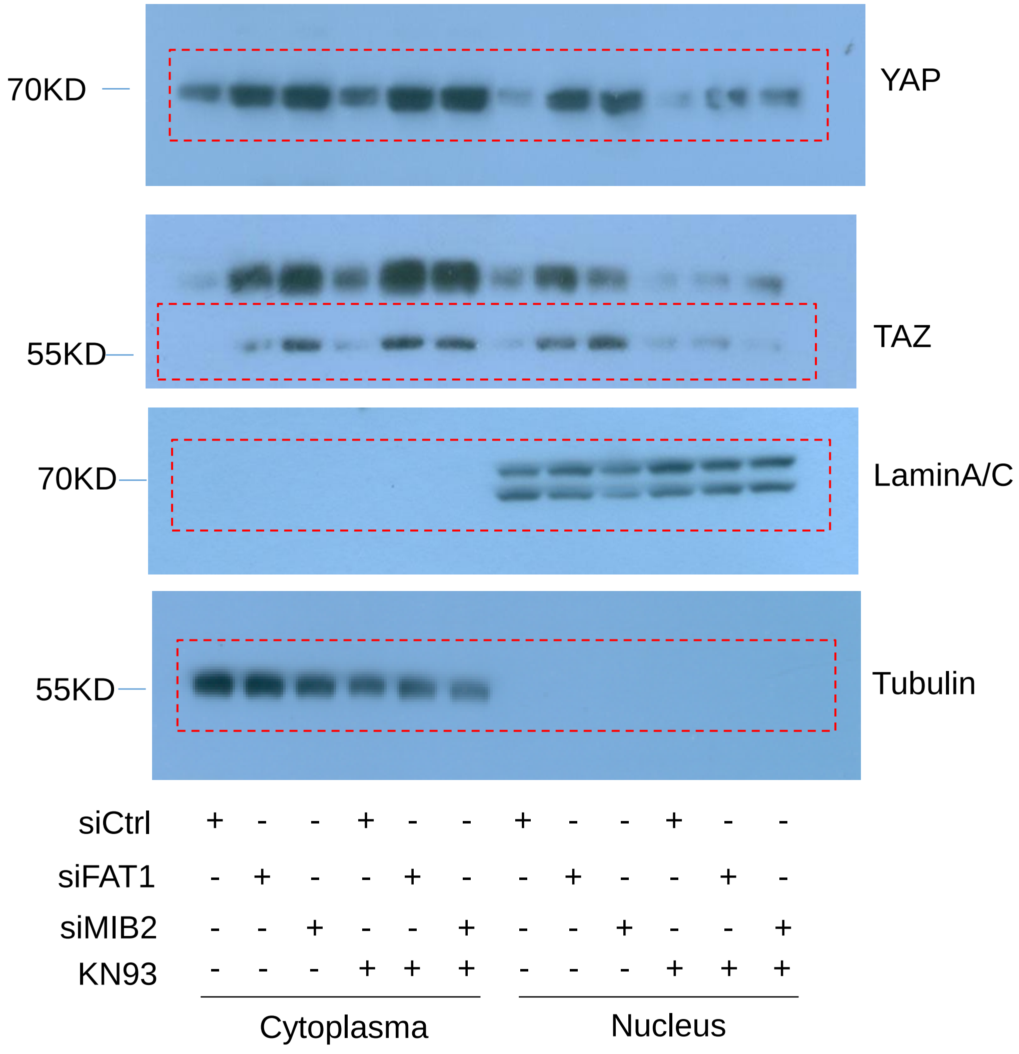

Fig. 5B

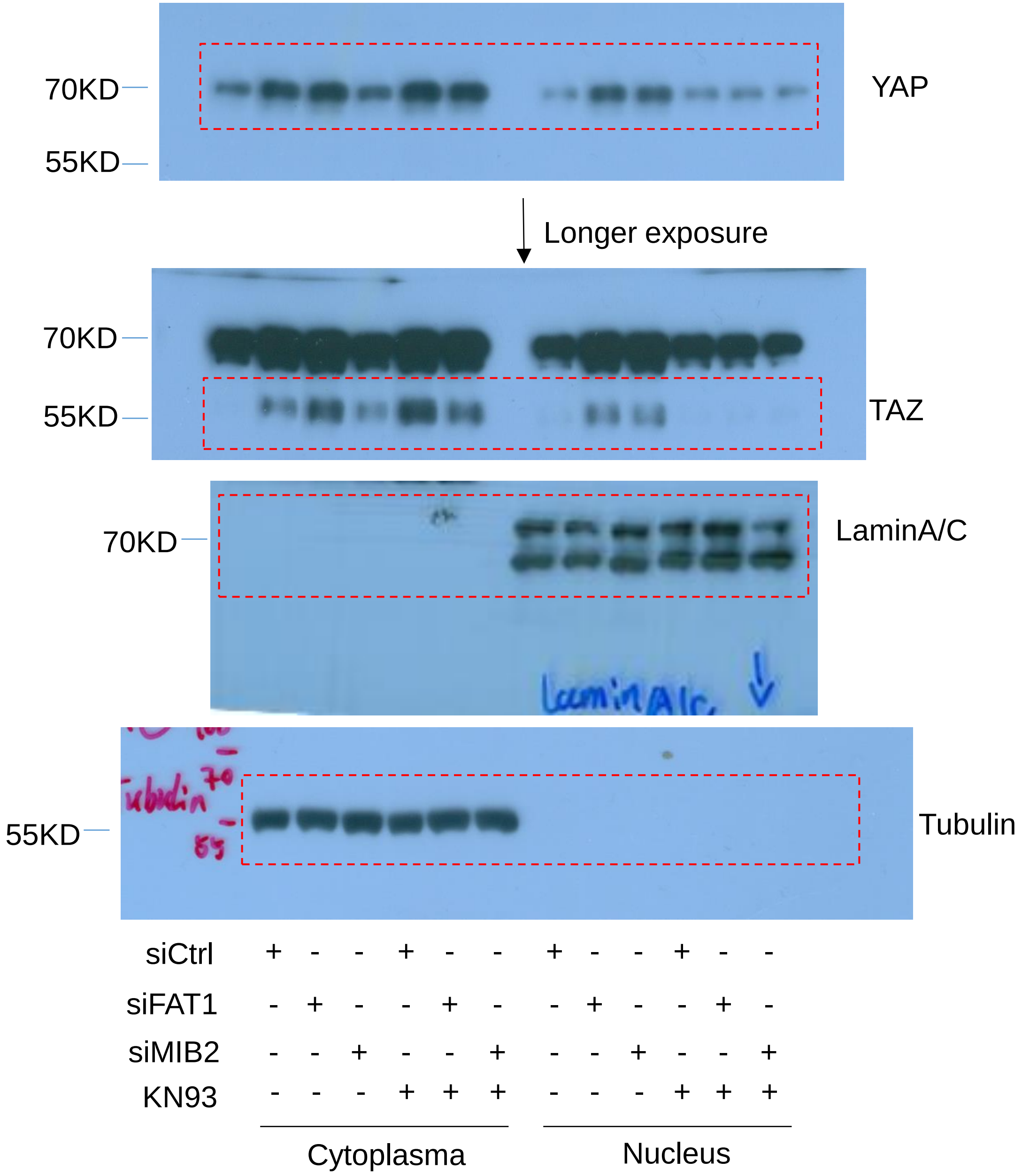

The blots shown here were obtained using film-based chemiluminescent detection.

Fig. 5C

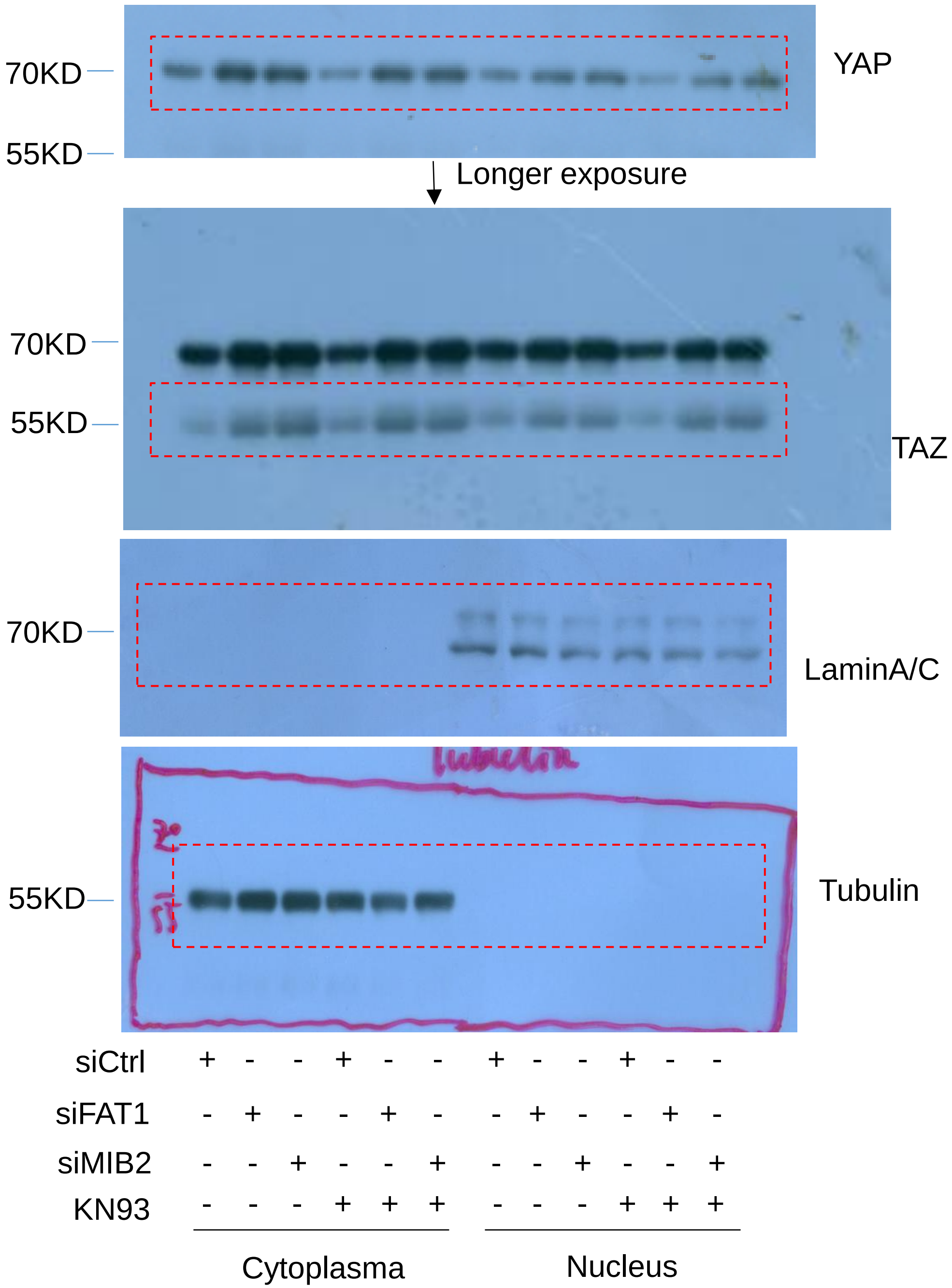

Fig. 5D

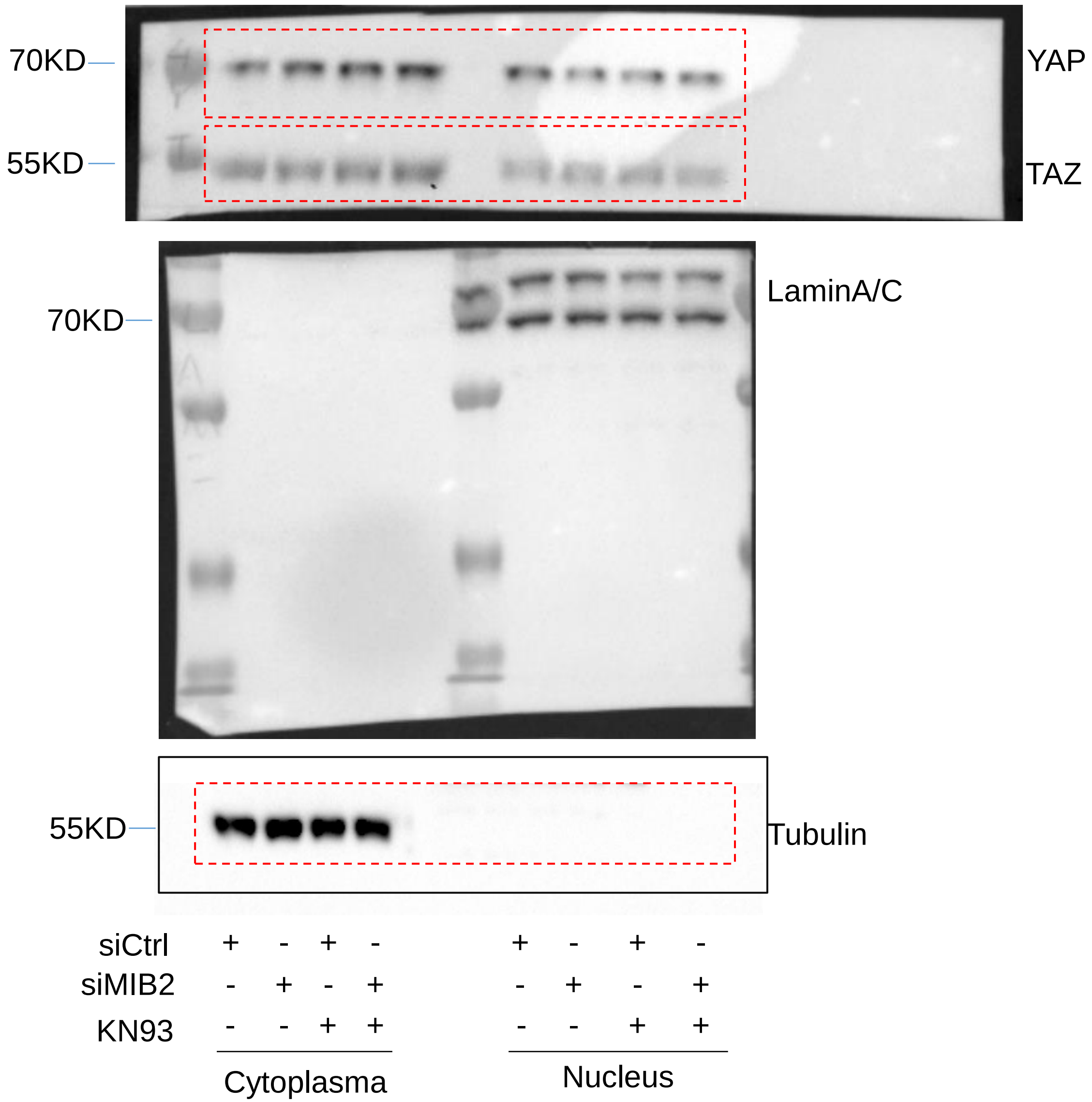

The blots shown in Fig. 5C here were obtained using film-based chemiluminescent detection and blots shown in Fig. 5D were obtained using ChemiDoc chemiluminescent Imaging.

Fig. 7C

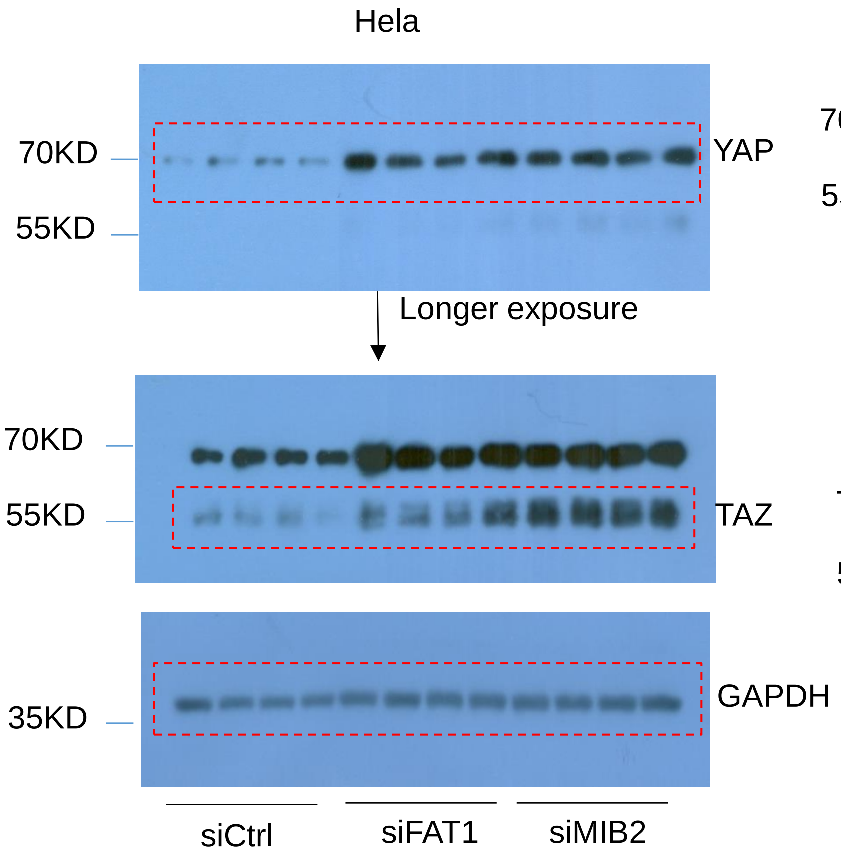

Fig. 7F

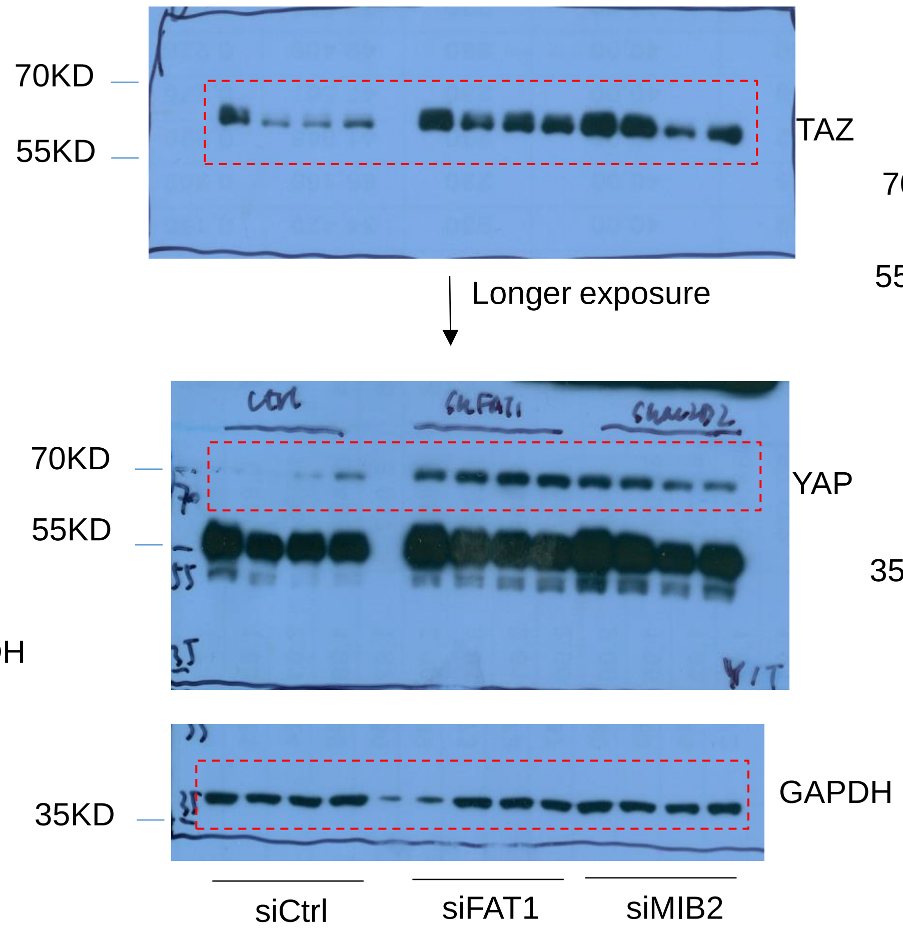

Fig. 7I

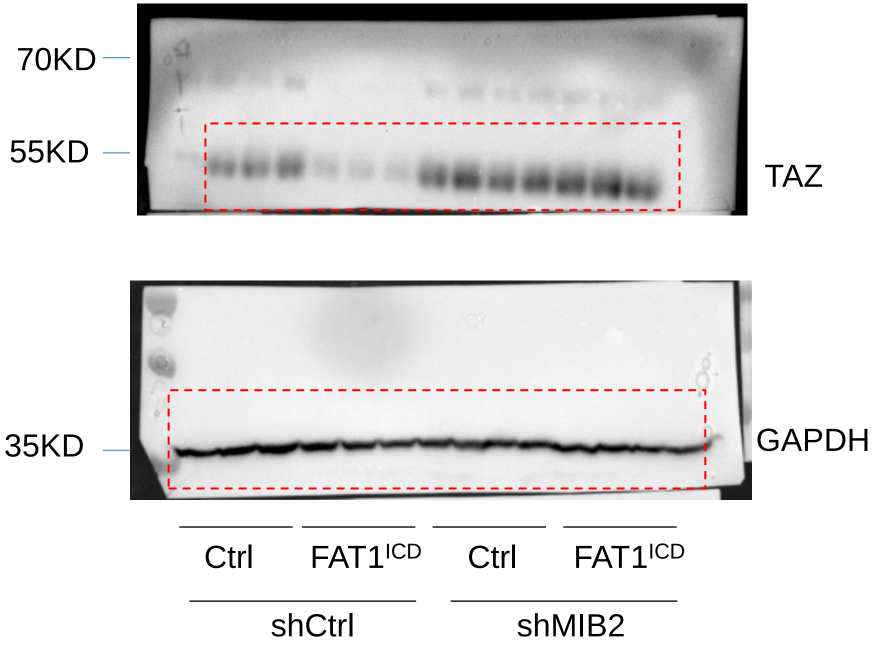

The blots shown in Fig. 7C and Fig. 7F here were obtained using film-based chemiluminescent detection and blots shown in Fig. 7I were obtained using ChemiDoc chemiluminescent Imaging.

S1 Fig

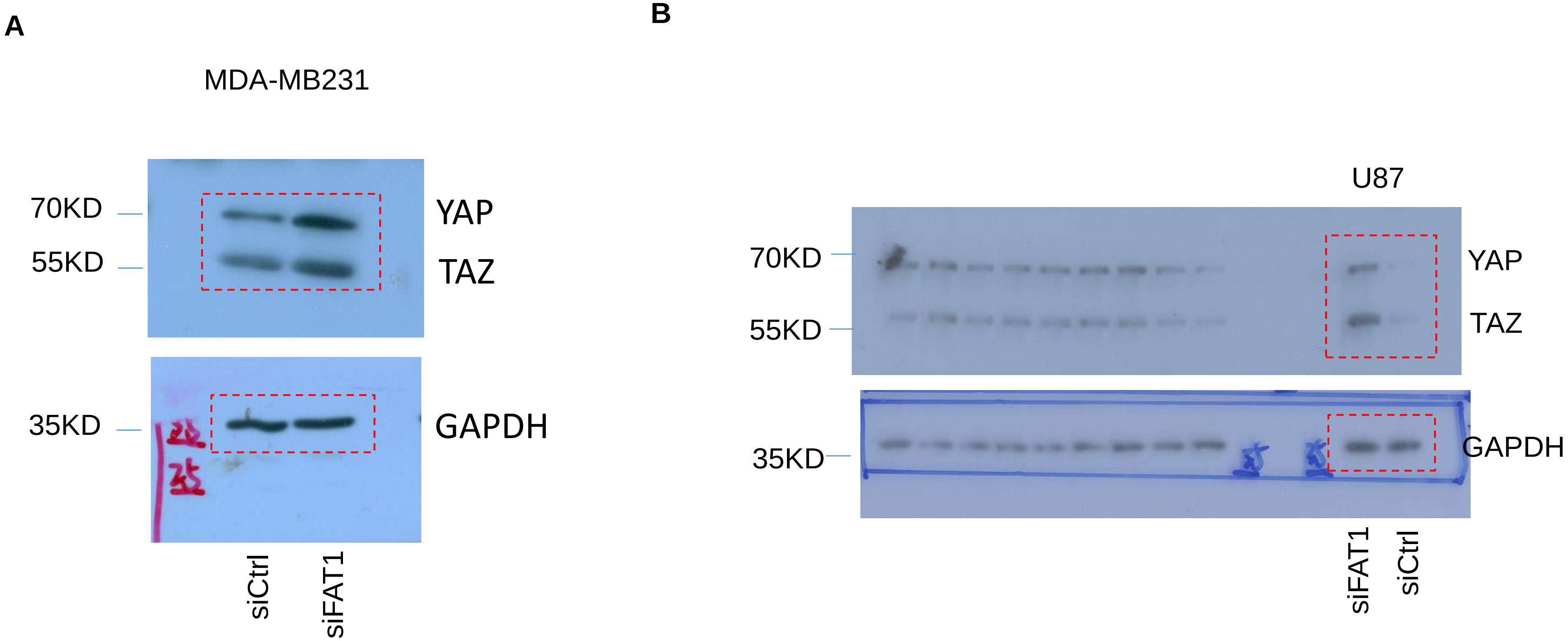

The blots shown here were obtained using film-based chemiluminescent detection.

S2 Fig.

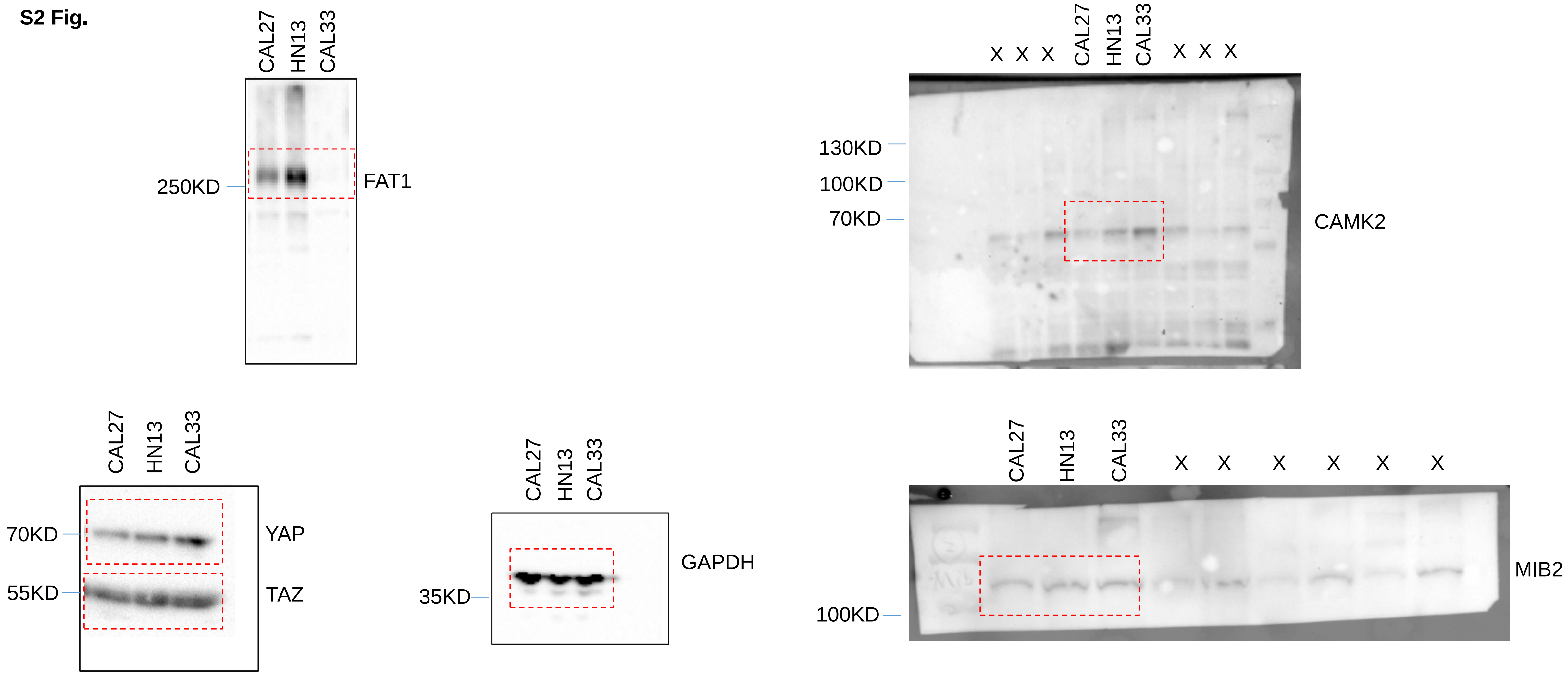

The blots shown here were obtained using ChemiDoc chemiluminescent Imaging.

S3A Fig.

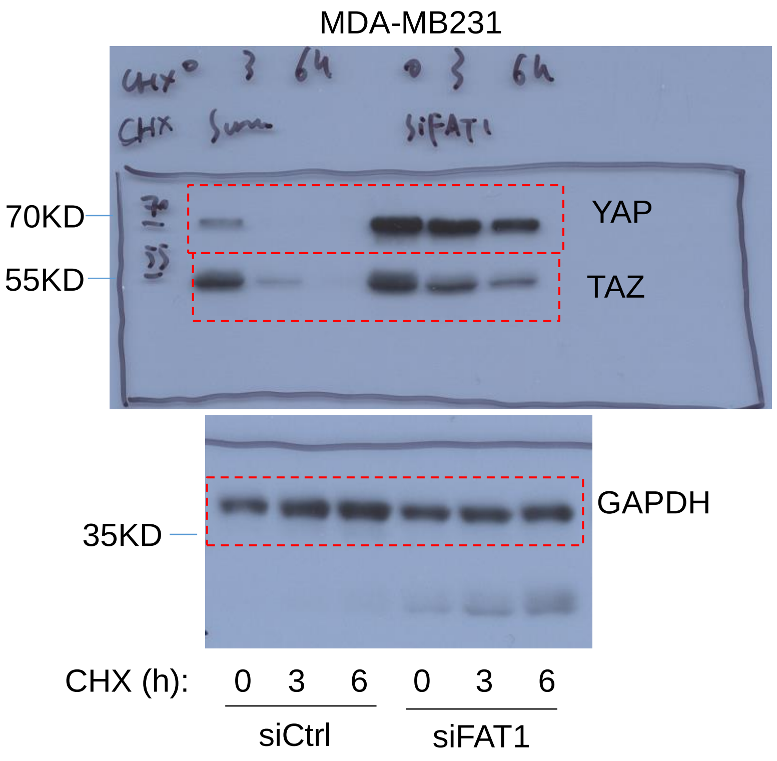

S3B Fig.

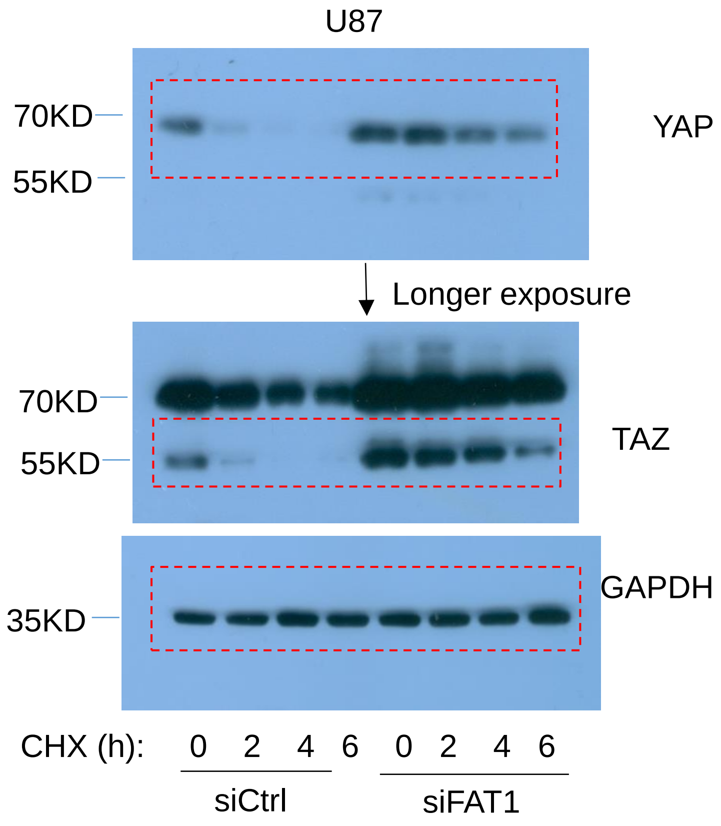

The blots shown here were obtained using film-based chemiluminescent detection.

S4A Fig.

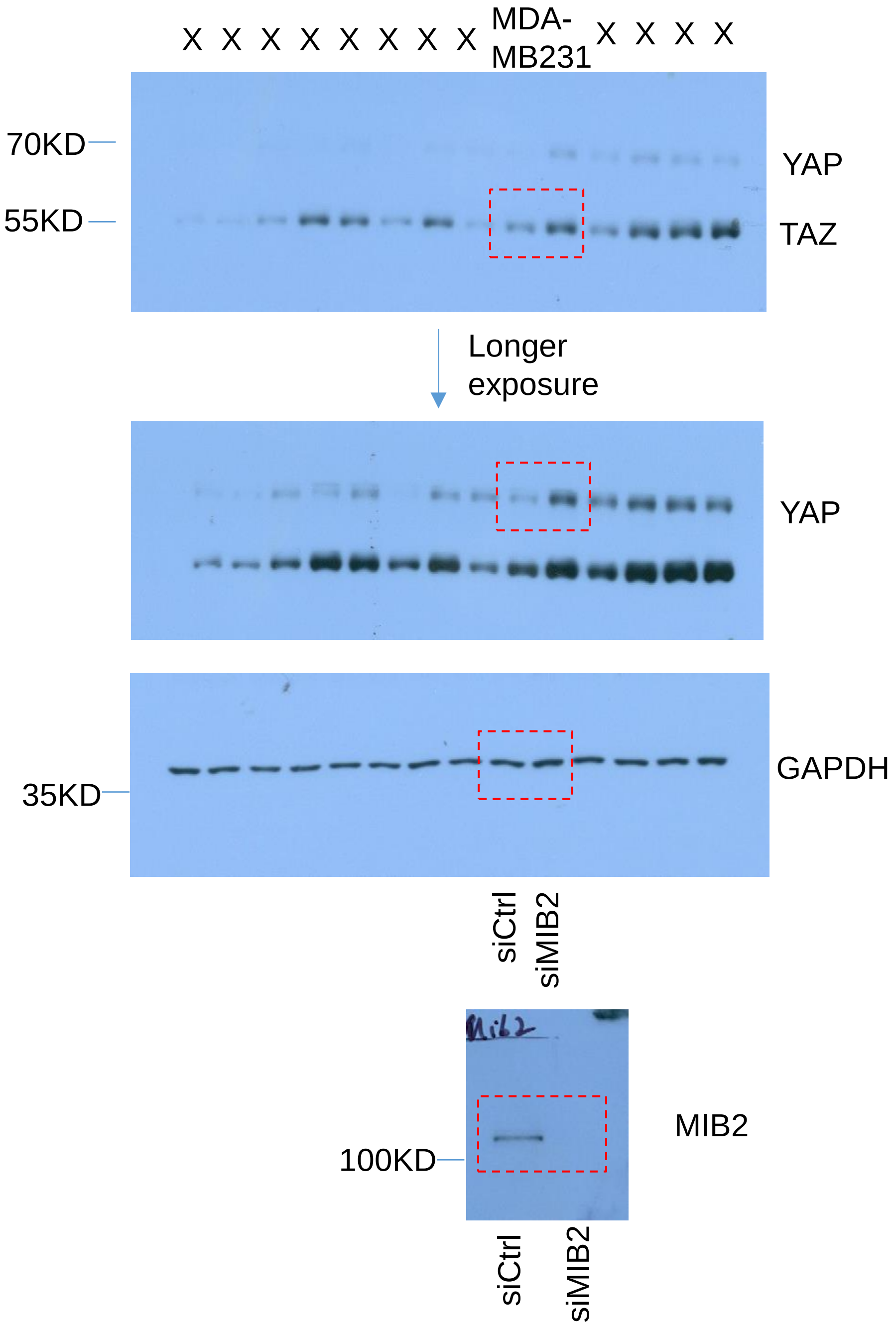

S4B Fig.

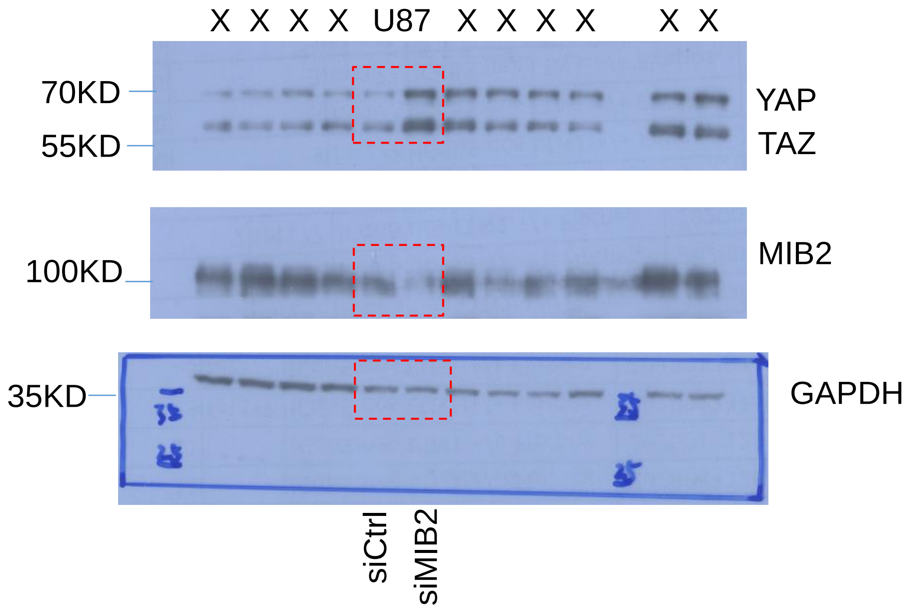

The blots shown here were obtained using film-based chemiluminescent detection.

S5A. Fig

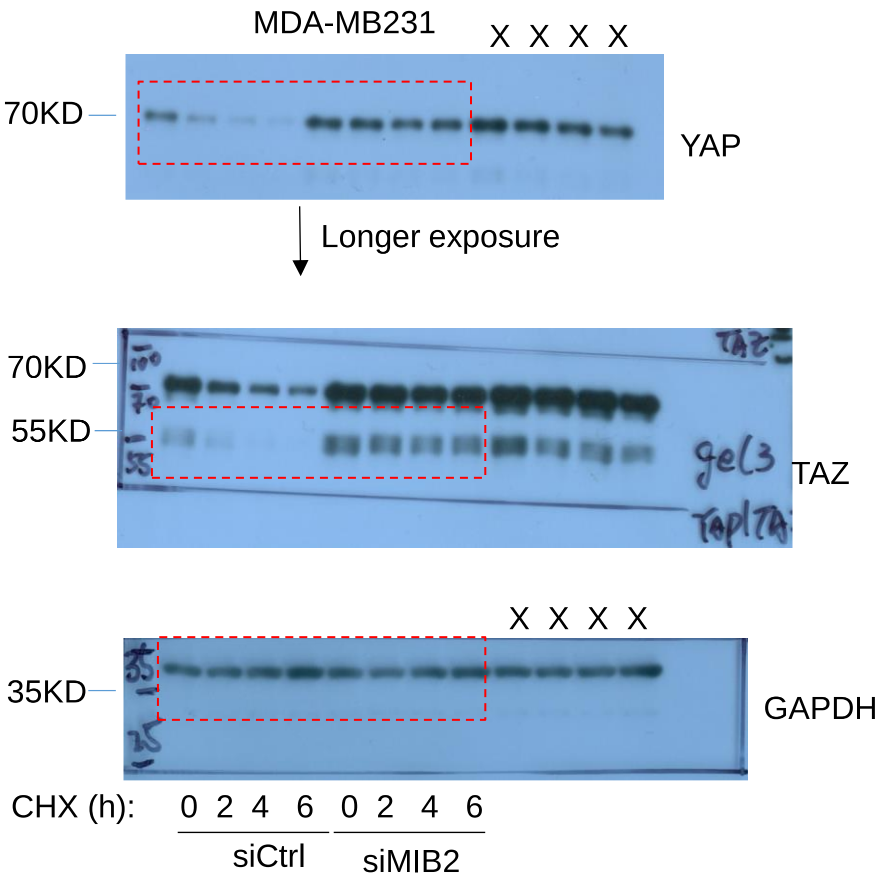

S5B. Fig

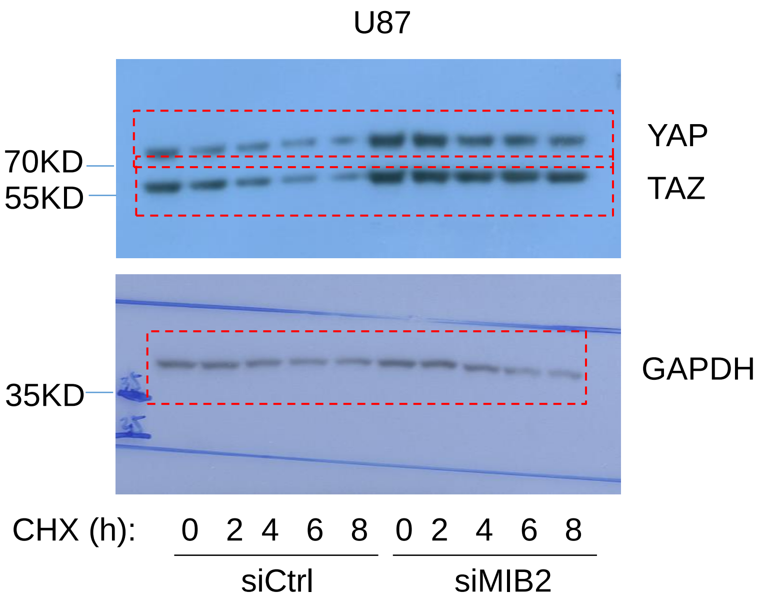

The blots shown here were obtained using film-based chemiluminescent detection.

S7A Fig.

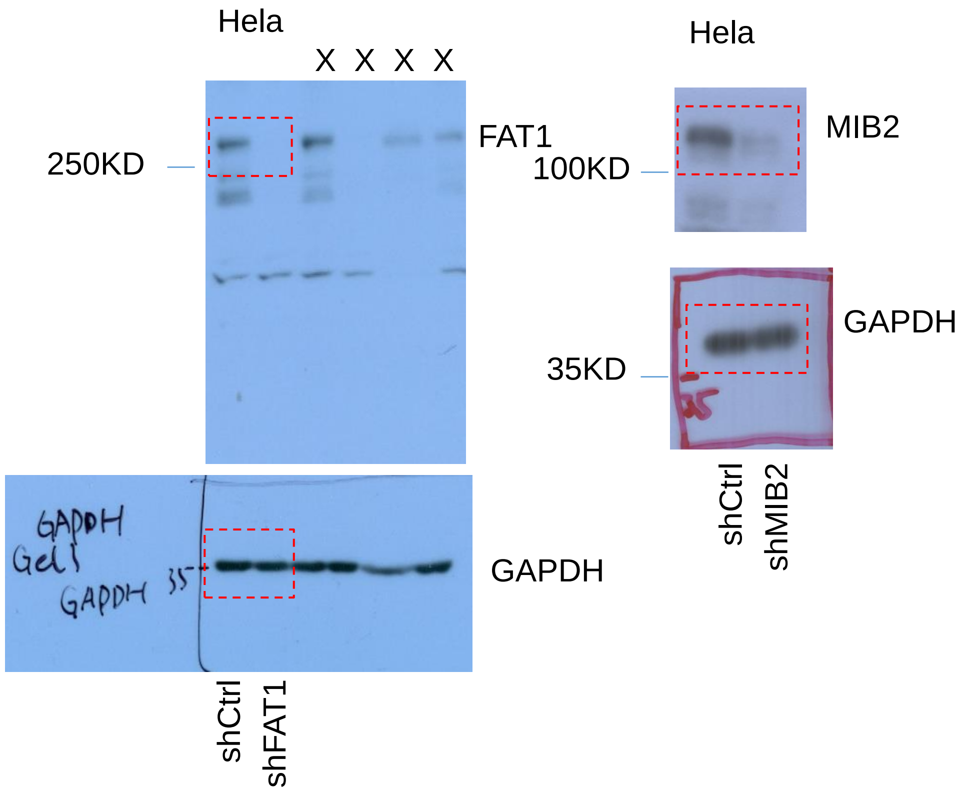

S7B Fig.

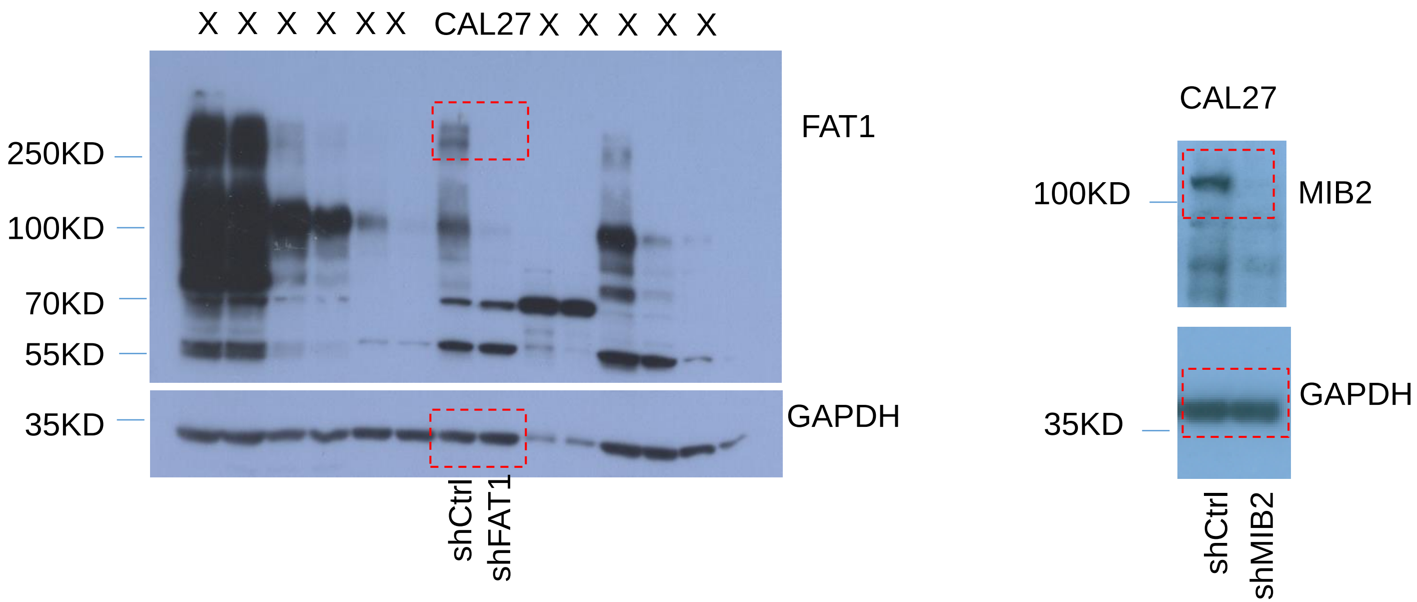

The blots shown in here were obtained using film-based chemiluminescent detection.

S7C Fig.

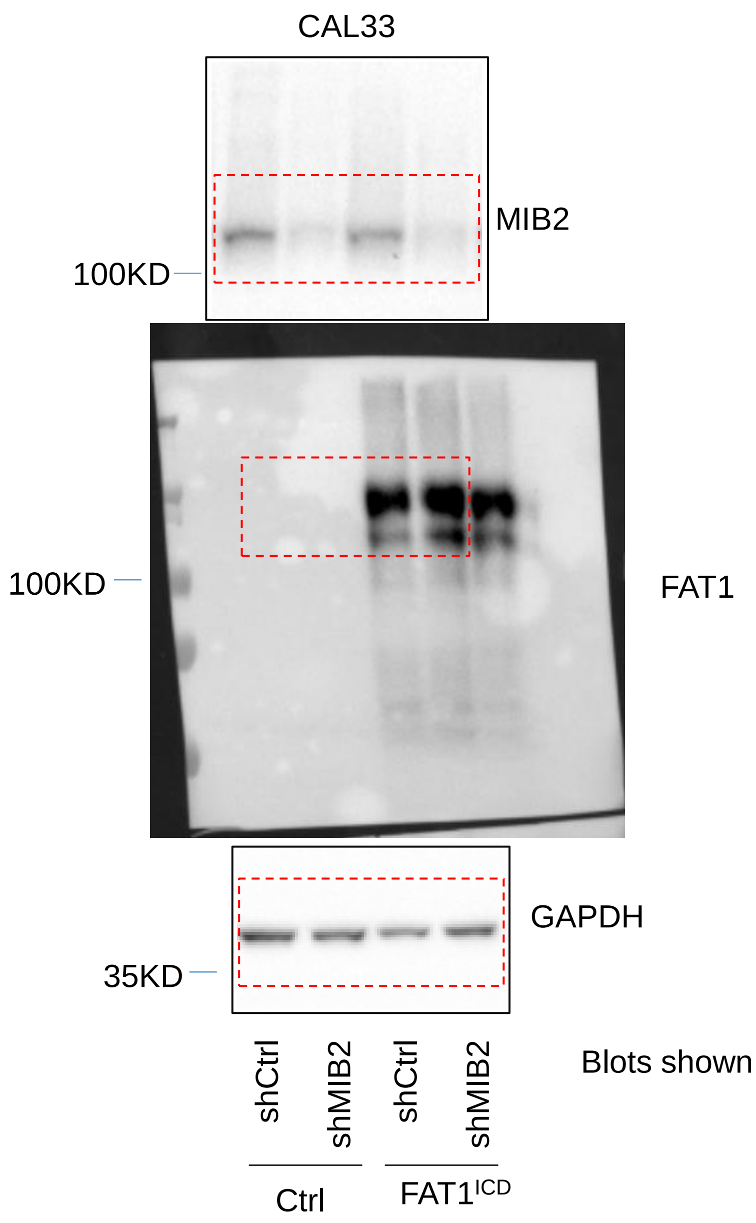

Blots shown in S7C Fig. were obtained using chemiDoc Chemiluminescent Imaging.
